# Causal relationship between linoleic acid and type 2 diabetes and glycemic traits: a bidirectional Mendelian randomization study

**DOI:** 10.3389/fendo.2023.1277153

**Published:** 2023-11-21

**Authors:** Hao Liang, Hai-Bo Mu, Fei-Hu Zhang, Wen-Qiang Li, Guo-Chen Li, Wen-Dong Li, Min Liang, Zeng-Lin He

**Affiliations:** ^1^ Shandong University of Traditional Chinese Medicine, College of Traditional Chinese Medicine, Jinan, China; ^2^ Centre for Emergency and Critical Care Medicine, Affiliated Hospital of Shandong University of Traditional Chinese Medicine, Jinan, China; ^3^ Department of Cardiovascular Disease, Tai’an Hospital of Traditional Chinese Medicine, Tai’an, China; ^4^ Department of Nephrology, Tai’an First People's Hospital, Tai’an, China; ^5^ School of Acupuncture and Massage, Shandong University of Traditional Chinese Medicine, Jinan, China

**Keywords:** linoleic acid, type 2 diabetes, glycemic traits, causal relationship, Mendelian randomization

## Abstract

**Objective:**

To investigate the causal relationships between linoleic acid and type 2 diabetes, and between linoleic acid and glycemic traits in European populations.

**Methods:**

This study employed a two-sample Mendelian randomization approach to infer causality between linoleic acid and type 2 diabetes, as well as between linoleic acid and glycemic traits, leveraging genetic variations. Data were sourced from genome-wide association study summary datasets. Random-effects inverse-variance weighted, weighted median, and MR-Egger methods were used for the two-sample Mendelian randomization analyses. Results were presented as odds ratios with a 95% confidence interval. Multiple sensitivity analyses were conducted to assess result robustness.

**Results:**

MR findings indicated a correlation between linoleic acid levels and the risk of type 2 diabetes, fasting blood glucose, and glycated hemoglobin (HbA1c), but not with fasting insulin. Specifically: type 2 diabetes (OR: 0.811, 95% CI: 0.688–0.956, *P*=0.013<0.05),fasting blood glucose (β_IVW): -0.056, 95% CI: (-0.091,-0.021), *P*=0.002< 0.0125), glycated hemoglobin (β_IVW: -0.032, 95% CI: (-0.048,-0.015), *P*=0.0002< 0.0125) and Fasting insulin (β_IVW: -0.024, 95% CI: (-0.056,-0.008), *P*=0.136 >0.05).Reverse MR analyses showed a correlation between type 2 diabetes and reduced levels of linoleic acid (β_IVW: -0.033, 95% CI: (-0.059,-0.006), *P*=0.014<0.05). Multiple sensitivity analyses also detected study heterogeneity but found no evidence of horizontal pleiotropy.

**Conclusion:**

High levels linoleic acid can reduce the risk of type 2 diabetes, fasting blood glucose, and glycated hemoglobin, but has no significant relation with fasting insulin. Type 2 diabetes can lower linoleic acid levels; however, no significant causal relationship was observed between the three glycemic traits and reduced levels of linoleic acid.

## 1 Introduction

Diabetes mellitus is a prevalent chronic metabolic disorder characterized by elevated blood glucose levels (1). Globally, the incidence of Type 2 Diabetes (T2D) has been on the rise. According to the International Diabetes Federation (IDF), as of 2021, an astonishing 536.6 million individuals, or 10.5% of the global population, were living with diabetes. This number is projected to soar to 783.2 million by 2045 (2). The global health burden imposed by diabetes has seen a significant uptick from 1990 to 2019 (3). T2D is a multifactorial condition marked by impaired pancreatic β-cell function and the onset of peripheral tissue insulin resistance, culminating in clinical manifestations (4). The etiology and causal associations of T2D may involve intricate interactions among genetic, behavioral, and environmental factors. These include sedentary lifestyles, poor dietary patterns, obesity, familial predisposition, advancing age, and even racial or ethnic attributes (5). T2D increases the risk for an array of diabetes-related complications such as cardiovascular diseases, renal anomalies, ocular abnormalities, microvascular disorders (6), and premature mortality (7). Consequently, a deep understanding of factors related to the genesis of T2D becomes pivotal to devise proactive strategies aiming to prevent and mitigate the adverse outcomes of this ailment.

Linoleic Acid (LA) is the predominant Ω-6 polyunsaturated fatty acid (PUFA), constituting about 80-90% of the overall dietary PUFA composition (8). The relationship between LA and T2D has sparked an ongoing discourse, encompassing diverse perspectives and academic debates. A meta-analysis synthesizing multiple cohort studies has corroborated the intimate association between LA and T2D (9). Various meta-analyses have probed into the pivotal role of LA in the pathogenesis of T2D. Consolidated findings elucidate a strong association between increased dietary LA intake and elevated physiological levels of LA, both of which are inversely correlated with the incidence of T2D. These compelling outcomes suggest that LA could potentially serve as a crucial protective factor against this metabolic disorder (10, 11). Nonetheless, the current evidence falls short of offering ample validation to endorse significant alterations in the intake of long-chain Ω-3 fatty acids, α-LA, Ω-6 fats, or overall PUFAs concerning their impact on glucose metabolism or diabetes risk (12). It’s hypothesized that Ω-6 PUFAs have a tight nexus with the onset of hyperinsulinemia, positioning Ω-6 PUFA as a distinguishable biomarker for hyperinsulinemia rather than exerting protective or detrimental effects on T2D (13). Moreover, certain studies have also indicated a correlation between LA and enhanced susceptibility to T2D (14). At present, most studies delving into the causal relationship between LA and T2D are grounded in observational and cohort research. However, to establish a definitive causality, rigorous randomized controlled trials are warranted. It’s noteworthy that executing such trials could be cost-prohibitive or unfeasible.

In Mendelian randomization(MR) studies, genetic variation adheres to the principle of random gene assortment, analogous to randomized controlled trials. MR serves as a potent tool in epidemiological research, leveraging genetic variants as instruments to elucidate causal associations between exposures and outcomes. By effectively sidestepping pitfalls of reverse causation and confounding factors, this methodological approach preserves the integrity of study findings, ensuring robust causal inferences (15). In traditional observational studies, describing causal relationships is challenging due to the potential presence of confounders and reverse causation. However, using genetic variants as instrumental variables (IVs), Mendelian randomization analysis offers a precise avenue to scrutinize and quantify these causal links.To ensure the reliability of such analyses, three fundamental assumptions must be satisfied: (1) IVs robustly correlate with the exposure, (2) they remain unaffected by confounders that could disrupt the relationship between exposure and outcome, and (3) they influence the outcome solely through their direct impact on exposure. In a comprehensive investigation of the effects of linoleic acid in the context of T2D, this study employed a two-sample MR analysis using data from genome-wide association study (GWAS). The primary objective was to discern the intricate causal relationship between LA levels in the blood and T2D, along with its associated glycemic traits.

## 2 Materials and methods

### 2.1 Study design and data sources

**Figure 1:**
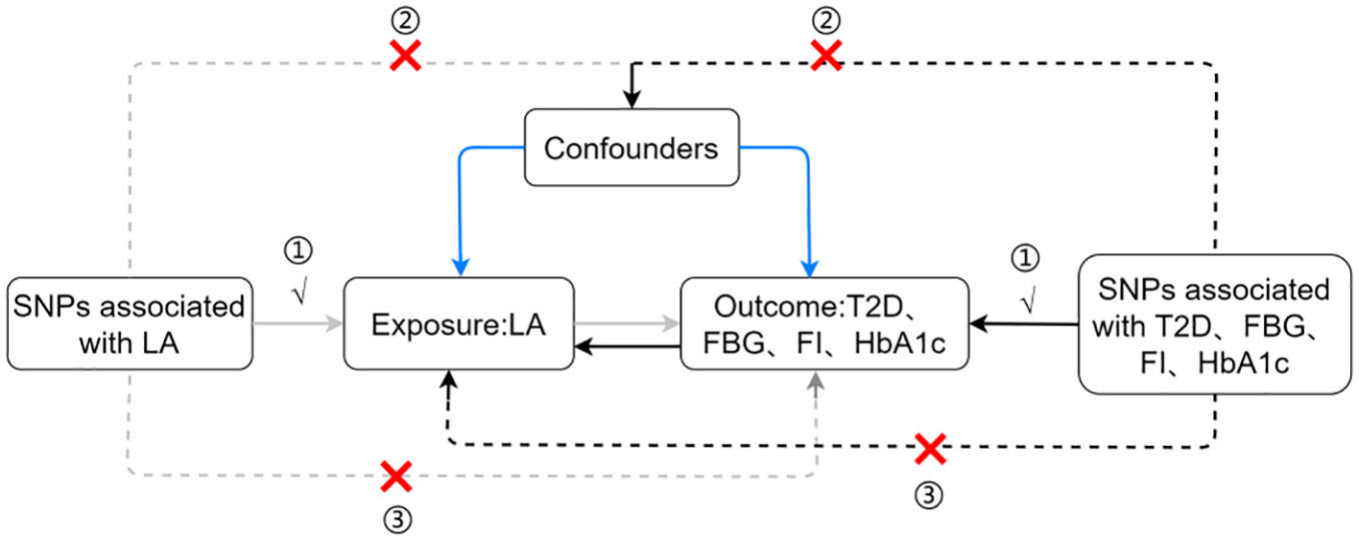
Assumptions and study design flowchart of the MR study. LA, linoleic acid; T2D, Type 2 Diabetes; FBG, fasting blood glucose; FI, fasting insulin.

This study employed a complex and academic research design, incorporating two-sample MR analyses, to meticulously investigate the intricate causal relationships among circulating LA concentrations and T2D, fasting blood glucose, fasting insulin, and glycated hemoglobin (HbA1c). The study rigorously scrutinized three key assumptions: (1) a profound and highly significant correlation exists between the IVs and circulating LA levels, (2) the IVs are unrelated to confounding factors, and (3) the IVs have no effect on T2D, fasting blood glucose, fasting insulin, and HbA1c, influencing the outcomes solely through its impact on LA. The primary MR investigated the causal relationship between circulating LA as the exposure variable and T2D along with its glycemic traits as outcome variables. Simultaneously, reverse causal relationships were explored by treating T2D and glycemic traits as exposure variables and circulating LA levels as the outcome variable (see **Figure 1**).

The GWAS data used in this study were sourced from the IEU OpenGWAS database, which provides an extensive range of comprehensive genetic information. A thorough exposition of this can be found in **Supplementary Table 1**, where a detailed narrative is provided.

### 2.2 IVs selection

We implemented a series of comprehensive quality control procedures to ensure the inclusion of reliable and robust genetic IVs. First, we identified single nucleotide polymorphisms (SNPs) associated with the exposure using the established genome-wide significance threshold (P< 5 × 10^(-8)), based on the original GWAS data. Subsequently, we clumped linkage disequilibrium, grounding our approach in European ancestry referential data (1000 Genomes Project, r^2^ = 0.001, clump window = 10,000 kb). This step ensured the independence of all selected SNPs while effectively mitigating potential confounding from linkage disequilibrium. To alleviate the impact of weak instrument bias, we applied an F-statistic threshold ( $\text{F}-\text{statistics}={\text{β}}^{2}/{\text{se}}^{2}$ ) (16) greater than 10 for all exposure factors. To exclude any distortions caused by strand or allele coding, palindromic SNPs were carefully excluded from the analysis. To circumvent potential pleiotropic effects, we utilized the PhenoScanner database (http://www.phenoscanner.medschl.cam.ac.uk/) as a tool to screen for IVs associated with confounding factors or risk factors.

### 2.3 Statistical analysis

We executed complex two-sample MR analyses in the R version 4.2.2 environment, using the TwoSampleMR 0.5.6 package, including inverse variance weighted (IVW) (17), weighted median (18), and MR-Egger (19). The primary method employed in the MR analysis was IVW, as results derived from the IVW method are generally considered the most robust when all IVs are valid (20). Additionally, the Weighted Median and MR-Egger served as secondary analysis methods for reference in our study. For continuous outcome variables, we represented the results with β and 95% confidence intervals(CI). For binary outcome variables, we transformed the effect estimates into odds ratios (OR), serving as evaluation metrics describing the causal relationships among variables. The OR values, along with their precisely calculated 95% confidence intervals (CI), endowed our study results with scientific rigor and robustness, thereby enhancing the overall quality and reliability of the research. Horizontal pleiotropic effects may occur when IVs associated with the exposure have effects on the outcomes beyond the direct scope of the exposure. To assess the presence of horizontal pleiotropy, we used the MR-Egger intercept test, where a significance threshold (*P*< 0.05) detection indicates potential pleiotropic influences, emphasizing the necessity for cautious interpretation of the obtained results. Furthermore, we employed Cochran’s Q statistic to investigate heterogeneity, with statistically significant heterogeneity (*P*< 0.05) detected among the included studies indicating their heterogeneity. To visually display these results, we used funnel plots. When adopting the “leave-one-out” approach (21), we sequentially excluded each independent IV and calculated the combined effect of the remaining IVs to verify result reliability. To address the issue of multiple comparisons among the four groups, we implemented Bonferroni correction (corrected P-value: 0.05/4 = 0.0125). A calculated *P*-value less than 0.0125 was classified as statistically significant, considering the adjustment for multiple comparisons. Furthermore, *P*-values between 0.0125 and 0.05 were considered suggestive of a causal relationship.

## 3 Results

### 3.1 Selection of IVs

The characteristics of carefully selected SNPs are elaborated in **Supplementary Tables 2–9**. Each selected SNP perfectly meets the selection criteria specified in the methods section. Furthermore, the IVs strength tests unequivocally demonstrate that there is no vulnerability to biased results, as all SNPs displayed an F-statistic value exceeding the threshold of 10.

### 3.2 Causal relationship between circulating levels of LA on T2D and glycemic traits.

The genetic predictive results for LA on T2D and glycemic traits are presented **Supplementary Figure 1**. Using IVW as the primary analysis method, the results reveal that linoleic acid is associated with a risk of T2D occurrence [OR: 0.811, 95% CI: 0.688–0.956, *P*=0.013] and fasting blood glucose concentration [β_IVW: -0.056, 95% CI: (-0.091,-0.021), *P*=0.002], with both the Weighed median and MR-Egger demonstrating the same causal relationship. However, MR-Egger did not find similar results; the results indicate that glycated hemoglobin [β_IVW: -0.032, 95% CI: (-0.048,-0.015), *P*=0.0002] exhibits a negative correlation, with both Weighed median and MR-Egger showing the same causal relationship. The results also demonstrate that linoleic acid has no significant causal relationship with fasting insulin [β_IVW: -0.024, 95% CI: (-0.056,-0.008), *P*=0.136], with MR-Egger and Weighed median similarly reflecting the same causal relationship, as shown in **Supplementary Figure 2**.

### 3.3 Reverse MR

The genetic prediction results of LA for T2D and glycemic traits are shown in **Supplementary Figure 3**. Analysis mainly conducted using IVW method indicates that T2D is associated with reduced concentrations of LA [β_IVW: -0.033, 95% CI: (-0.059,-0.006), *P*=0.014]. The findings are consistent with weighted median, although not significant in MR-Egger analysis, as seen in **Supplementary Figure 4**. There were no significant associations between fasting plasma glucose, fasting insulin, and glycated hemoglobin with levels of LA (**Supplementary Figure 3**), including fasting blood glucose [β_IVW: -0.077, 95% CI: (-0.249,-0.094), *P*=0.375], fasting insulin [β_IVW: -0.16, 95% CI: (-0.394,0.074), *P*=0.181], and HbA1c [β_IVW: 0.136, 95% CI: (-0.075,0.347), *P*=0.207]. The results are in agreement with the remaining weighted median and MR-Egger, although the weighted median method for glycated hemoglobin and LA levels shows a significant association between the two [β_IVW:0.213, 95% CI: (0.097,-0.330), *P*=0.0003], as depicted in **Supplementary Figure 4**.

### 3.4 Sensitivity analysis in MR

We conducted heterogeneity tests and horizontal pleiotropy tests on the results of MR analysis and reverse MR analysis for two sets of samples. Cochran’s Q test revealed significant heterogeneity across all study outcomes (*P*<0.05) (see **Table 1**; **Supplementary Figures 5, 6**). Therefore, a random effects model was utilized in the MR analysis to ensure the reliability of the results. Tests for horizontal pleiotropy showed no apparent trend toward zero for MR-Egger intercept term, and all study *P*-values were greater than 0.05 (see **Table 1**), indicating no underlying horizontal pleiotropy. Additionally, by employing a “leave-one-out” sensitivity analysis, where each SNP was excluded one by one and the MR analysis was repeated, the results obtained were consistent with the analysis including all SNPs. As illustrated in **Supplementary Figures 7, 8**, the determined causal relationships did not exhibit significant differences.

**Table 1:** Summary of Sensitivity Analysis Results.

MR analysis	SNP(n)	Heterogeneith test			Pleiotropy test		
		Q	Q_df	Q-pval	MR-Eggerintercept	SE	P
LA-T2D	28	220	27	2.91175E-32	-0.0054	0.0119	0.655
LA-fasting blood glucose	49	466	48	7.1208E-70	-0.0017	0.0022	0.455
LA-fasting insulin	49	292	48	8.235E-37	-0.0004	0.00205	0.83995
LA-HbA1c	49	183	48	1.03497E-17	0.000985475	0.001063011	0.358631551
T2D-LA	115	573	114	2.0631E-62	-0.00118	0.002209	0.5942
Fasting glucose-LA	64	728	63	5.681E-114	-0.00072	0.003681	0.8455
Fasting insulin-LA	38	241	37	6.5623E-32	0.007944	0.005912	0.187407
HbA1c-LA	72	692	71	8.95023E-103	-0.00416	0.003178	0.19465

## 4 Discussion

Numerous previous studies have employed MR to determine the causal relationship between LA and various diseases, including but not limited to cardiovascular diseases, sleep apnea, and sepsis (22–24). However, current evidence regarding linoleic acid and T2D primarily stems from cohort studies, with results and views exhibiting evident contradictions and conflicts (10–14). Thus, a thorough exploration of the explicit association between the two will assist in early identification of potential patients, and this work becomes especially crucial for early monitoring and effective prevention and treatment.

This study is the first to apply bidirectional MR analysis to deeply investigate the impact of LA on the occurrence of T2D and glycemic traits. It aims to reveal the causal relationships among LA, T2D, and glycemic traits. The evidence obtained clearly indicates significant correlations between LA and T2D prevalence, along with various glycemic traits. High levels LA not only reduces the incidence of T2D but effectively lowers fasting blood glucose and glycated hemoglobin levels. Simultaneously, reverse MR analysis suggests a correlation between T2D and reduced levels of LA, but no substantive association exists between fasting glucose, fasting insulin, and glycated hemoglobin with circulating LA levels.

Meta-analysis from prospective cohort studies undeniably demonstrates a close correlation between increased dietary intake of LA and increased body concentration of LA, significantly reducing the likelihood of contracting T2D (11). A comprehensive integrated analysis covering 20 prospective longitudinal studies (10) clearly shows that, compared to arachidonic acid, increased LA concentration is strongly associated with reduced susceptibility to T2D, consistent with MR causal inference results. Additionally, this research reveals that high levels of LA can improve glucose and insulin resistance, fully aligning with causal inference results: first, glycated hemoglobin can be used as an indicator to assess average plasma glucose levels over the previous three months (25); second, the Homeostatic Model Assessment for Insulin Resistance (HOMA-IR) index is used to assess insulin resistance, calculated as fasting insulin (μU/mL) × fasting blood glucose (mmol/L)/22.5 (26), and we can deduce that LA may improve insulin resistance. In reverse causal inference analysis, we found a correlation between T2D and reduced levels of LA, a view consistent with previous research (27).

Although the exact causal mechanism by which LA levels reduce the risk of developing T2D occurrence risk has not been fully elucidated, scholars have proposed several explanations. LA, a vital component of cell membranes, can alter membrane fluidity and regulate insulin sensitivity when integrated into phospholipids (28). High levels of LA can increase total and High Molecular Weight (HMW) adiponectin concentrations (29), A recent study showed a positive correlation between lipocalin levels and insulin sensitivity, lipocalin improves insulin sensitivity through its upregulation of IRS-2 in the liver and can lower blood glucose (30, 31). This may be a mechanism by which LA reduces the risk of developing T2D.Additionally, LA is considered a PUFA in most Western diets, found in plant oils, nuts, and seeds (32). LA is metabolized in various tissues by delta-6 desaturase to form gamma-linolenic acid (GLA), which is then rapidly elongated to dihomo-gamma-linolenic acid (DGLA). DGLA can be further desaturated by delta-5 desaturase to form arachidonic acid (AA). However, human delta-5 desaturase activity is limited, with only a portion of DGLA being converted to AA (33, 34). Metabolites of DGLA, such as prostaglandin E1, can enhance insulin action (35). AA is also a precursor to various important bioactive lipid mediators, including series 2 prostaglandins (such as PGE2, PGD2, PGF2α, and PGI2) (36). PGE2 not only enhances insulin sensitivity but also increases muscle glycolysis (37). PGD2 also regulates insulin, with PGD2 in white adipose tissue primarily produced by hematopoietic PGD synthase in macrophages, causing macrophages to polarize from an inflammatory M1 state to an anti-inflammatory M2 state. This polarization of macrophages correlates positively with adipose insulin sensitivity (38). These results suggest that LA metabolites PGE1 and PGE2 may improve adipose insulin resistance by enhancing insulin sensitivity, and PGD2 may do so by regulating macrophage polarization, thereby reducing the risk of T2D. As for the mechanism of T2D reducing LA levels, it’s not entirely clear, but possible mechanisms include a significant increase in the abundance of Lactobacillus in the gut microbiome of T2D patients (39). In the gastrointestinal tract, Lactobacillus promotes the transformation of LA, thus reducing the gastrointestinal uptake of LA and lowering circulating LA levels. This phenomenon may explain a potential mechanism for reduced LA concentration in T2D patients.

These findings presented in this study may hold significant clinical implications. The prevalence of T2D is experiencing a sharp increase globally (2), underscoring the necessity for extensive preventive and therapeutic measures. We found that maintaining high levels LA might benefit in reducing the risk of T2D onset. In all major guidelines, diet is the cornerstone of prevention and treatment (40). Therefore, from a healthcare professional perspective, it may be worth considering recommending more LA-rich foods or supplements to high-risk populations for T2D, especially those with genetic susceptibility. However, it should be noted that these findings are solely based on genetic analysis, so further research is needed to validate the clinical efficacy of these recommendations.

This MR study exhibits the following advantages. Firstly, it employs genetic variations as IVs to infer the causal relationships between LA and T2D occurrence risk and glycemic traits. This approach significantly minimizes the potential influences of confounding factors and reverse causality, both of which are common challenges in traditional observational studies (41–43). Secondly, the study leverages large-scale genomic data provided by the IEU summary database, notably enhancing the applicability and robustness of the research findings. Thirdly, the utilization of public datasets and open-source software further bolsters the transparency and reproducibility of the study. Fourthly, this method allows for the assessment of the degree of causal effects, the results of which may have substantial and lasting impacts on clinical and public health decisions. Overall, this MR study offers vital insights into the causal relationships between LA and the risk of T2D onset and glycemic traits.

Despite our study’s strengths, there are certain acknowledged limitations. Firstly, our reliance on GWAS data involving only European ancestry populations hampers the generalizability of our findings to other groups, imposing a constraint on the study in terms of population diversity. Secondly, our research lacks adequate sensitivity analysis to assess the possibility of horizontal pleiotropy, even though we used the MR-Egger intercept test and found no clear evidence of horizontal pleiotropy in our statistical analysis. Thirdly, we observed some heterogeneity in the results. Nevertheless, the random-effects IVW remains the primary analytical method, effectively controlling for the influence of summary data heterogeneity. Fourthly, our sole reliance on genetic-level evidence limits further observational studies and mediation analyses to validate the specific regulatory mechanisms of the causal relationship between LA, T2D, and glycemic traits. Fifthly, we only studied linear causal relationships, as LA levels were treated as a continuous variable. Therefore, future research needs to encompass broader, more diverse populations, spanning different ancestries and cultures, and further conduct non-linear MR analysis to delve into the potential non-linear effects of LA levels on T2D and glycemic traits.

## 5 Conclusion

Overall, our study is groundbreaking, employing a bidirectional sample MR analysis to explore the causal relationships between LA, T2D, and glycemic traits. The results of this MR study validate the existence of a bidirectional causal link between glycemic traits and T2D. We found a significant negative correlation between high levels LA and susceptibility to T2D, and the occurrence of T2D seems to lead to a decrease in LA levels. Additionally, we observed a negative correlation between high levels LA and fasting blood glucose, as well as glycated hemoglobin levels. However, the complex relationship between LA and T2D, the association between LA and glycemic traits, and the potential mechanisms governing these relationships, require further in-depth exploration through original research. Considering the adverse consequences associated with T2D and the causal relationships identified in our study, we recommend increasing LA intake as a primary preventive measure for T2D.

## Data availability statement

The original contributions presented in the study are included in the article/**Supplementary Material**. Further inquiries can be directed to the corresponding author.

## Author contributions

HL: Data curation, Formal Analysis, Software, Visualization, Writing – original draft, Writing – review & editing. H-BM: Data curation, Writing – original draft and Writing – review & editing. F-HZ: Writing – review & editing. W-QL: Funding acquisition, Methodology, Writing – review & editing. G-CL: Funding acquisition, Writing – review & editing. W-DL: Validation, Writing – review & editing. ML: Supervision, Writing – review & editing. Z-LH: Writing – review & editing.
