# Supplementary material for: Causal relationship between linoleic acid and type 2 diabetes and glycemic traits: a bidirectional Mendelian randomization study
Source: Front Endocrinol (Lausanne). 2023 Nov 21;14:1277153. doi: 10.3389/fendo.2023.1277153 (PMC10703485; doi:10.3389/fendo.2023.1277153)
Supplement: Supplementary file 1 [file DataSheet_1.docx]

Supplementary Material

## Supplementary Table

**Supplementary Table 1. Summary of genome-wide association studies (GWAS) datasets in our study.**

| **Phenotype** | **GWAS ID** | **Year** | **Population** | **Sample size** | **No. of cases (Binary trait)** | **PMID** |
| --- | --- | --- | --- | --- | --- | --- |
| Linoleic acid | met-d-LA | 2020 | European | 114,999 | NA | NA |
| Type 2 diabetes | ebi-a-GCST006867 | 2018 | European | 655,666 | 61,714 | 30054458 |
| Fasting blood glucose | ebi-a-GCST90002232 | 2021 | European | 200,622 | NA | 34059833 |
| Fasting insulin | ebi-a-GCST90002238 | 2021 | European | 151,013 | NA | 34059833 |
| HbA1c | ebi-a-GCST90002244 | 2021 | European | 146,806 | NA | 34059833 |

HbA1c, glycated hemoglobin;

**Supplementary Table 2 Mendelian randomization analysis of linoleic acid and type 2 diabetes.**

| SNP | effect_allele | other_allele | eaf | beta | se | P-value | mr_keep | F(β2)/(se2) |
| --- | --- | --- | --- | --- | --- | --- | --- | --- |
| rs1002687 | A | G | 0.644748 | 0.0868452 | 0.0042312 | 1.80011E-97 | TRUE | 421.2738787 |
| rs1065853 | T | G | 0.080578 | -0.188865 | 0.00746999 | 1.9011E-143 | TRUE | 639.238502 |
| rs1081105 | C | A | 0.027624 | 0.120669 | 0.0123858 | 8.19974E-23 | TRUE | 94.91684664 |
| rs10838724 | T | G | 0.368821 | -0.0262357 | 0.00424528 | 1.6E-10 | TRUE | 38.19202035 |
| rs112875651 | A | G | 0.392346 | -0.0510959 | 0.00420654 | 5.00035E-35 | TRUE | 147.5441723 |
| rs11789603 | T | C | 0.108831 | 0.0454099 | 0.00651811 | 1.69981E-12 | TRUE | 48.53530021 |
| rs13108218 | G | A | 0.615417 | -0.0327027 | 0.00419342 | 5.90065E-16 | TRUE | 60.81777063 |
| rs13217434 | C | G | 0.259322 | 0.0433091 | 0.00471025 | 4.30031E-20 | TRUE | 84.54158799 |
| rs141469619 | G | A | 0.01014 | 0.123842 | 0.0213968 | 3.79997E-10 | TRUE | 33.49949496 |
| rs142158911 | A | G | 0.116705 | -0.0915875 | 0.00634525 | 1.20005E-48 | TRUE | 208.3410908 |
| rs1461729 | G | A | 0.899221 | 0.0703705 | 0.00673534 | 5.40008E-26 | TRUE | 109.159673 |
| rs174564 | G | A | 0.347013 | 0.0842755 | 0.00424412 | 5.10035E-88 | TRUE | 394.3005347 |
| rs186696265 | T | C | 0.014358 | -0.227024 | 0.0170817 | 2.19989E-41 | TRUE | 176.6368707 |
| rs1883711 | C | G | 0.031191 | 0.089775 | 0.0119031 | 3.69999E-15 | TRUE | 56.88407013 |
| rs2378390 | A | G | 0.140871 | -0.0324917 | 0.00584433 | 7.00003E-09 | TRUE | 30.90832062 |
| rs247617 | A | C | 0.323625 | 0.0506744 | 0.00433307 | 6.4998E-33 | TRUE | 136.768417 |
| rs261290 | C | T | 0.654653 | -0.089118 | 0.00426927 | 7.89951E-99 | TRUE | 435.7361779 |
| rs2740488 | C | A | 0.265321 | -0.0482851 | 0.00459865 | 1E-25 | TRUE | 110.2466893 |
| rs2986164 | A | G | 0.535856 | -0.0266721 | 0.00442883 | 5E-10 | TRUE | 36.26906844 |
| rs3011437 | G | T | 0.294371 | 0.0357926 | 0.0044745 | 1.29987E-16 | TRUE | 63.98784279 |
| rs34232196 | T | C | 0.245325 | -0.0305851 | 0.00474221 | 8.30042E-11 | TRUE | 41.59663943 |
| rs35599691 | A | G | 0.577598 | 0.0350233 | 0.00475488 | 6.59933E-12 | TRUE | 54.25440517 |
| rs35633876 | T | G | 0.481841 | -0.0344611 | 0.00406887 | 2.39994E-17 | TRUE | 71.73162068 |
| rs36018387 | T | C | 0.105092 | -0.0442699 | 0.00662522 | 6.4003E-12 | TRUE | 44.64948701 |
| rs4008004 | A | C | 0.221842 | 0.0309046 | 0.00489606 | 1.89998E-10 | TRUE | 39.84306969 |
| rs4299376 | T | G | 0.676279 | -0.0387395 | 0.00433864 | 2.49977E-19 | TRUE | 79.72614963 |
| rs4665972 | C | T | 0.604642 | -0.0513405 | 0.00416674 | 1.29987E-35 | TRUE | 151.8194397 |
| rs4704210 | C | G | 0.374212 | 0.0511244 | 0.00418591 | 1.99986E-35 | TRUE | 149.1683464 |
| rs4947302 | T | C | 0.064792 | 0.0459621 | 0.00824431 | 4.79999E-10 | TRUE | 31.08072204 |
| rs534417 | G | A | 0.875005 | 0.0359986 | 0.00610468 | 2.5E-09 | TRUE | 34.77326058 |
| rs55747707 | A | G | 0.203615 | -0.0449899 | 0.00504536 | 1.50003E-18 | TRUE | 79.51439084 |
| rs56322906 | A | G | 0.035157 | -0.0883014 | 0.0110068 | 2.90001E-15 | TRUE | 64.35955435 |
| rs58542926 | T | C | 0.074383 | -0.109822 | 0.00772171 | 7.50067E-48 | TRUE | 202.2794308 |
| rs602633 | G | T | 0.78295 | 0.0531806 | 0.00490259 | 7.50067E-28 | TRUE | 117.667172 |
| rs633695 | G | A | 0.292348 | 0.0697 | 0.00446965 | 1.99986E-54 | TRUE | 243.1747751 |
| rs6471717 | A | G | 0.663091 | -0.0308556 | 0.00429616 | 1.39991E-13 | TRUE | 51.58305948 |
| rs6602911 | T | C | 0.360079 | 0.0238048 | 0.00421825 | 1.40001E-08 | TRUE | 31.84670051 |
| rs6882345 | A | G | 0.632863 | 0.0441266 | 0.00419712 | 1.99986E-26 | TRUE | 110.5345784 |
| rs693 | A | G | 0.520883 | 0.0614441 | 0.00405281 | 3.40017E-54 | TRUE | 229.851788 |
| rs7139079 | A | G | 0.592501 | -0.0259545 | 0.00413291 | 4.79999E-10 | TRUE | 39.43786878 |
| rs740516 | G | C | 0.151132 | -0.03364 | 0.00568287 | 1.89998E-09 | TRUE | 35.04100323 |
| rs7707394 | A | G | 0.357106 | 0.0316688 | 0.00422065 | 3.90032E-14 | TRUE | 56.29949971 |
| rs7750288 | G | A | 0.285011 | 0.0265974 | 0.0044814 | 1.09999E-09 | TRUE | 35.22499578 |
| rs77960347 | G | A | 0.013239 | 0.248604 | 0.0176915 | 1.10002E-45 | TRUE | 197.4635364 |
| rs7816447 | C | T | 0.100848 | -0.0487086 | 0.00671655 | 8.19974E-13 | TRUE | 52.5918914 |
| rs79429216 | A | G | 0.012713 | 0.146817 | 0.0180405 | 1.59993E-16 | TRUE | 66.23012141 |
| rs9302635 | C | T | 0.182464 | -0.0283511 | 0.00523184 | 3.40001E-08 | TRUE | 29.36506184 |
| rs9304381 | T | C | 0.818434 | 0.0628471 | 0.00525856 | 1E-33 | TRUE | 142.8357166 |
| rs964184 | C | G | 0.867229 | -0.145606 | 0.00596821 | 1.9999E-136 | TRUE | 595.2101804 |

Abbreviation: SNP, single nucleotide polymorphism; SE, standard error; EAF, effect allele frequency;

Supplementary Table 3 Mendelian randomization analysis of linoleic acid and  fasting blood glucose.

| SNP | effect_allele | other_allele | eaf | beta | se | pval | mr_keep | F(β2)/(se2) |
| --- | --- | --- | --- | --- | --- | --- | --- | --- |
| rs1002687 | A | G | 0.644748 | 0.0868452 | 0.0042312 | 1.80011E-97 | TRUE | 421.2738787 |
| rs1065853 | T | G | 0.080578 | -0.188865 | 0.00746999 | 1.9011E-143 | TRUE | 639.238502 |
| rs1081105 | C | A | 0.027624 | 0.120669 | 0.0123858 | 8.19974E-23 | TRUE | 94.91684664 |
| rs10838724 | T | G | 0.368821 | -0.0262357 | 0.00424528 | 1.6E-10 | TRUE | 38.19202035 |
| rs112875651 | A | G | 0.392346 | -0.0510959 | 0.00420654 | 5.00035E-35 | TRUE | 147.5441723 |
| rs11789603 | T | C | 0.108831 | 0.0454099 | 0.00651811 | 1.69981E-12 | TRUE | 48.53530021 |
| rs13108218 | G | A | 0.615417 | -0.0327027 | 0.00419342 | 5.90065E-16 | TRUE | 60.81777063 |
| rs13217434 | C | G | 0.259322 | 0.0433091 | 0.00471025 | 4.30031E-20 | TRUE | 84.54158799 |
| rs141469619 | G | A | 0.01014 | 0.123842 | 0.0213968 | 3.79997E-10 | TRUE | 33.49949496 |
| rs142158911 | A | G | 0.116705 | -0.0915875 | 0.00634525 | 1.20005E-48 | TRUE | 208.3410908 |
| rs1461729 | G | A | 0.899221 | 0.0703705 | 0.00673534 | 5.40008E-26 | TRUE | 109.159673 |
| rs174564 | G | A | 0.347013 | 0.0842755 | 0.00424412 | 5.10035E-88 | TRUE | 394.3005347 |
| rs186696265 | T | C | 0.014358 | -0.227024 | 0.0170817 | 2.19989E-41 | TRUE | 176.6368707 |
| rs1883711 | C | G | 0.031191 | 0.089775 | 0.0119031 | 3.69999E-15 | TRUE | 56.88407013 |
| rs2378390 | A | G | 0.140871 | -0.0324917 | 0.00584433 | 7.00003E-09 | TRUE | 30.90832062 |
| rs247617 | A | C | 0.323625 | 0.0506744 | 0.00433307 | 6.4998E-33 | TRUE | 136.768417 |
| rs261290 | C | T | 0.654653 | -0.089118 | 0.00426927 | 7.89951E-99 | TRUE | 435.7361779 |
| rs2740488 | C | A | 0.265321 | -0.0482851 | 0.00459865 | 1E-25 | TRUE | 110.2466893 |
| rs2986164 | A | G | 0.535856 | -0.0266721 | 0.00442883 | 5E-10 | TRUE | 36.26906844 |
| rs3011437 | G | T | 0.294371 | 0.0357926 | 0.0044745 | 1.29987E-16 | TRUE | 63.98784279 |
| rs34232196 | T | C | 0.245325 | -0.0305851 | 0.00474221 | 8.30042E-11 | TRUE | 41.59663943 |
| rs35599691 | A | G | 0.577598 | 0.0350233 | 0.00475488 | 6.59933E-12 | TRUE | 54.25440517 |
| rs35633876 | T | G | 0.481841 | -0.0344611 | 0.00406887 | 2.39994E-17 | TRUE | 71.73162068 |
| rs36018387 | T | C | 0.105092 | -0.0442699 | 0.00662522 | 6.4003E-12 | TRUE | 44.64948701 |
| rs4008004 | A | C | 0.221842 | 0.0309046 | 0.00489606 | 1.89998E-10 | TRUE | 39.84306969 |
| rs4299376 | T | G | 0.676279 | -0.0387395 | 0.00433864 | 2.49977E-19 | TRUE | 79.72614963 |
| rs4665972 | C | T | 0.604642 | -0.0513405 | 0.00416674 | 1.29987E-35 | TRUE | 151.8194397 |
| rs4704210 | C | G | 0.374212 | 0.0511244 | 0.00418591 | 1.99986E-35 | TRUE | 149.1683464 |
| rs4947302 | T | C | 0.064792 | 0.0459621 | 0.00824431 | 4.79999E-10 | TRUE | 31.08072204 |
| rs534417 | G | A | 0.875005 | 0.0359986 | 0.00610468 | 2.5E-09 | TRUE | 34.77326058 |
| rs55747707 | A | G | 0.203615 | -0.0449899 | 0.00504536 | 1.50003E-18 | TRUE | 79.51439084 |
| rs56322906 | A | G | 0.035157 | -0.0883014 | 0.0110068 | 2.90001E-15 | TRUE | 64.35955435 |
| rs58542926 | T | C | 0.074383 | -0.109822 | 0.00772171 | 7.50067E-48 | TRUE | 202.2794308 |
| rs602633 | G | T | 0.78295 | 0.0531806 | 0.00490259 | 7.50067E-28 | TRUE | 117.667172 |
| rs633695 | G | A | 0.292348 | 0.0697 | 0.00446965 | 1.99986E-54 | TRUE | 243.1747751 |
| rs6471717 | A | G | 0.663091 | -0.0308556 | 0.00429616 | 1.39991E-13 | TRUE | 51.58305948 |
| rs6602911 | T | C | 0.360079 | 0.0238048 | 0.00421825 | 1.40001E-08 | TRUE | 31.84670051 |
| rs6882345 | A | G | 0.632863 | 0.0441266 | 0.00419712 | 1.99986E-26 | TRUE | 110.5345784 |
| rs693 | A | G | 0.520883 | 0.0614441 | 0.00405281 | 3.40017E-54 | TRUE | 229.851788 |
| rs7139079 | A | G | 0.592501 | -0.0259545 | 0.00413291 | 4.79999E-10 | TRUE | 39.43786878 |
| rs740516 | G | C | 0.151132 | -0.03364 | 0.00568287 | 1.89998E-09 | TRUE | 35.04100323 |
| rs7707394 | A | G | 0.357106 | 0.0316688 | 0.00422065 | 3.90032E-14 | TRUE | 56.29949971 |
| rs7750288 | G | A | 0.285011 | 0.0265974 | 0.0044814 | 1.09999E-09 | TRUE | 35.22499578 |
| rs77960347 | G | A | 0.013239 | 0.248604 | 0.0176915 | 1.10002E-45 | TRUE | 197.4635364 |
| rs7816447 | C | T | 0.100848 | -0.0487086 | 0.00671655 | 8.19974E-13 | TRUE | 52.5918914 |
| rs79429216 | A | G | 0.012713 | 0.146817 | 0.0180405 | 1.59993E-16 | TRUE | 66.23012141 |
| rs9302635 | C | T | 0.182464 | -0.0283511 | 0.00523184 | 3.40001E-08 | TRUE | 29.36506184 |
| rs9304381 | T | C | 0.818434 | 0.0628471 | 0.00525856 | 1E-33 | TRUE | 142.8357166 |
| rs964184 | C | G | 0.867229 | -0.145606 | 0.00596821 | 1.9999E-136 | TRUE | 595.2101804 |

Abbreviation: SNP, single nucleotide polymorphism; SE, standard error; EAF, effect allele frequency;

Supplementary Table 4 Mendelian randomization analysis of linoleic acid and fasting insulin.

| SNP | effect_allele | other_allele | eaf | beta | se | pval | mr_keep | F(β2)/(se2) |
| --- | --- | --- | --- | --- | --- | --- | --- | --- |
| rs1002687 | A | G | 0.644748 | 0.0868452 | 0.0042312 | 1.80011E-97 | TRUE | 421.2738787 |
| rs1065853 | T | G | 0.080578 | -0.188865 | 0.00746999 | 1.9011E-143 | TRUE | 639.238502 |
| rs1081105 | C | A | 0.027624 | 0.120669 | 0.0123858 | 8.19974E-23 | TRUE | 94.91684664 |
| rs10838724 | T | G | 0.368821 | -0.0262357 | 0.00424528 | 1.6E-10 | TRUE | 38.19202035 |
| rs112875651 | A | G | 0.392346 | -0.0510959 | 0.00420654 | 5.00035E-35 | TRUE | 147.5441723 |
| rs11789603 | T | C | 0.108831 | 0.0454099 | 0.00651811 | 1.69981E-12 | TRUE | 48.53530021 |
| rs13108218 | G | A | 0.615417 | -0.0327027 | 0.00419342 | 5.90065E-16 | TRUE | 60.81777063 |
| rs13217434 | C | G | 0.259322 | 0.0433091 | 0.00471025 | 4.30031E-20 | TRUE | 84.54158799 |
| rs141469619 | G | A | 0.01014 | 0.123842 | 0.0213968 | 3.79997E-10 | TRUE | 33.49949496 |
| rs142158911 | A | G | 0.116705 | -0.0915875 | 0.00634525 | 1.20005E-48 | TRUE | 208.3410908 |
| rs1461729 | G | A | 0.899221 | 0.0703705 | 0.00673534 | 5.40008E-26 | TRUE | 109.159673 |
| rs174564 | G | A | 0.347013 | 0.0842755 | 0.00424412 | 5.10035E-88 | TRUE | 394.3005347 |
| rs186696265 | T | C | 0.014358 | -0.227024 | 0.0170817 | 2.19989E-41 | TRUE | 176.6368707 |
| rs1883711 | C | G | 0.031191 | 0.089775 | 0.0119031 | 3.69999E-15 | TRUE | 56.88407013 |
| rs2378390 | A | G | 0.140871 | -0.0324917 | 0.00584433 | 7.00003E-09 | TRUE | 30.90832062 |
| rs247617 | A | C | 0.323625 | 0.0506744 | 0.00433307 | 6.4998E-33 | TRUE | 136.768417 |
| rs261290 | C | T | 0.654653 | -0.089118 | 0.00426927 | 7.89951E-99 | TRUE | 435.7361779 |
| rs2740488 | C | A | 0.265321 | -0.0482851 | 0.00459865 | 1E-25 | TRUE | 110.2466893 |
| rs2986164 | A | G | 0.535856 | -0.0266721 | 0.00442883 | 5E-10 | TRUE | 36.26906844 |
| rs3011437 | G | T | 0.294371 | 0.0357926 | 0.0044745 | 1.29987E-16 | TRUE | 63.98784279 |
| rs34232196 | T | C | 0.245325 | -0.0305851 | 0.00474221 | 8.30042E-11 | TRUE | 41.59663943 |
| rs35599691 | A | G | 0.577598 | 0.0350233 | 0.00475488 | 6.59933E-12 | TRUE | 54.25440517 |
| rs35633876 | T | G | 0.481841 | -0.0344611 | 0.00406887 | 2.39994E-17 | TRUE | 71.73162068 |
| rs36018387 | T | C | 0.105092 | -0.0442699 | 0.00662522 | 6.4003E-12 | TRUE | 44.64948701 |
| rs4008004 | A | C | 0.221842 | 0.0309046 | 0.00489606 | 1.89998E-10 | TRUE | 39.84306969 |
| rs4299376 | T | G | 0.676279 | -0.0387395 | 0.00433864 | 2.49977E-19 | TRUE | 79.72614963 |
| rs4665972 | C | T | 0.604642 | -0.0513405 | 0.00416674 | 1.29987E-35 | TRUE | 151.8194397 |
| rs4704210 | C | G | 0.374212 | 0.0511244 | 0.00418591 | 1.99986E-35 | TRUE | 149.1683464 |
| rs4947302 | T | C | 0.064792 | 0.0459621 | 0.00824431 | 4.79999E-10 | TRUE | 31.08072204 |
| rs534417 | G | A | 0.875005 | 0.0359986 | 0.00610468 | 2.5E-09 | TRUE | 34.77326058 |
| rs55747707 | A | G | 0.203615 | -0.0449899 | 0.00504536 | 1.50003E-18 | TRUE | 79.51439084 |
| rs56322906 | A | G | 0.035157 | -0.0883014 | 0.0110068 | 2.90001E-15 | TRUE | 64.35955435 |
| rs58542926 | T | C | 0.074383 | -0.109822 | 0.00772171 | 7.50067E-48 | TRUE | 202.2794308 |
| rs602633 | G | T | 0.78295 | 0.0531806 | 0.00490259 | 7.50067E-28 | TRUE | 117.667172 |
| rs633695 | G | A | 0.292348 | 0.0697 | 0.00446965 | 1.99986E-54 | TRUE | 243.1747751 |
| rs6471717 | A | G | 0.663091 | -0.0308556 | 0.00429616 | 1.39991E-13 | TRUE | 51.58305948 |
| rs6602911 | T | C | 0.360079 | 0.0238048 | 0.00421825 | 1.40001E-08 | TRUE | 31.84670051 |
| rs6882345 | A | G | 0.632863 | 0.0441266 | 0.00419712 | 1.99986E-26 | TRUE | 110.5345784 |
| rs693 | A | G | 0.520883 | 0.0614441 | 0.00405281 | 3.40017E-54 | TRUE | 229.851788 |
| rs7139079 | A | G | 0.592501 | -0.0259545 | 0.00413291 | 4.79999E-10 | TRUE | 39.43786878 |
| rs740516 | G | C | 0.151132 | -0.03364 | 0.00568287 | 1.89998E-09 | TRUE | 35.04100323 |
| rs7707394 | A | G | 0.357106 | 0.0316688 | 0.00422065 | 3.90032E-14 | TRUE | 56.29949971 |
| rs7750288 | G | A | 0.285011 | 0.0265974 | 0.0044814 | 1.09999E-09 | TRUE | 35.22499578 |
| rs77960347 | G | A | 0.013239 | 0.248604 | 0.0176915 | 1.10002E-45 | TRUE | 197.4635364 |
| rs7816447 | C | T | 0.100848 | -0.0487086 | 0.00671655 | 8.19974E-13 | TRUE | 52.5918914 |
| rs79429216 | A | G | 0.012713 | 0.146817 | 0.0180405 | 1.59993E-16 | TRUE | 66.23012141 |
| rs9302635 | C | T | 0.182464 | -0.0283511 | 0.00523184 | 3.40001E-08 | TRUE | 29.36506184 |
| rs9304381 | T | C | 0.818434 | 0.0628471 | 0.00525856 | 1E-33 | TRUE | 142.8357166 |
| rs964184 | C | G | 0.867229 | -0.145606 | 0.00596821 | 1.9999E-136 | TRUE | 595.2101804 |

Abbreviation: SNP, single nucleotide polymorphism; SE, standard error; EAF, effect allele frequency;

Supplementary Table 5 Mendelian randomization analysis of linoleic acid and glycosylated hemoglobin.

| SNP | effect_allele | other_allele | eaf | beta | se | pval | mr_keep | F(β2)/(se2) |
| --- | --- | --- | --- | --- | --- | --- | --- | --- |
| rs1002687 | A | G | 0.644748 | 0.0868452 | 0.0042312 | 1.80011E-97 | TRUE | 421.2738787 |
| rs1065853 | T | G | 0.080578 | -0.188865 | 0.00746999 | 1.9011E-143 | TRUE | 639.238502 |
| rs1081105 | C | A | 0.027624 | 0.120669 | 0.0123858 | 8.19974E-23 | TRUE | 94.91684664 |
| rs10838724 | T | G | 0.368821 | -0.0262357 | 0.00424528 | 1.6E-10 | TRUE | 38.19202035 |
| rs112875651 | A | G | 0.392346 | -0.0510959 | 0.00420654 | 5.00035E-35 | TRUE | 147.5441723 |
| rs11789603 | T | C | 0.108831 | 0.0454099 | 0.00651811 | 1.69981E-12 | TRUE | 48.53530021 |
| rs13108218 | G | A | 0.615417 | -0.0327027 | 0.00419342 | 5.90065E-16 | TRUE | 60.81777063 |
| rs13217434 | C | G | 0.259322 | 0.0433091 | 0.00471025 | 4.30031E-20 | TRUE | 84.54158799 |
| rs141469619 | G | A | 0.01014 | 0.123842 | 0.0213968 | 3.79997E-10 | TRUE | 33.49949496 |
| rs142158911 | A | G | 0.116705 | -0.0915875 | 0.00634525 | 1.20005E-48 | TRUE | 208.3410908 |
| rs1461729 | G | A | 0.899221 | 0.0703705 | 0.00673534 | 5.40008E-26 | TRUE | 109.159673 |
| rs174564 | G | A | 0.347013 | 0.0842755 | 0.00424412 | 5.10035E-88 | TRUE | 394.3005347 |
| rs186696265 | T | C | 0.014358 | -0.227024 | 0.0170817 | 2.19989E-41 | TRUE | 176.6368707 |
| rs1883711 | C | G | 0.031191 | 0.089775 | 0.0119031 | 3.69999E-15 | TRUE | 56.88407013 |
| rs2378390 | A | G | 0.140871 | -0.0324917 | 0.00584433 | 7.00003E-09 | TRUE | 30.90832062 |
| rs247617 | A | C | 0.323625 | 0.0506744 | 0.00433307 | 6.4998E-33 | TRUE | 136.768417 |
| rs261290 | C | T | 0.654653 | -0.089118 | 0.00426927 | 7.89951E-99 | TRUE | 435.7361779 |
| rs2740488 | C | A | 0.265321 | -0.0482851 | 0.00459865 | 1E-25 | TRUE | 110.2466893 |
| rs2986164 | A | G | 0.535856 | -0.0266721 | 0.00442883 | 5E-10 | TRUE | 36.26906844 |
| rs3011437 | G | T | 0.294371 | 0.0357926 | 0.0044745 | 1.29987E-16 | TRUE | 63.98784279 |
| rs34232196 | T | C | 0.245325 | -0.0305851 | 0.00474221 | 8.30042E-11 | TRUE | 41.59663943 |
| rs35599691 | A | G | 0.577598 | 0.0350233 | 0.00475488 | 6.59933E-12 | TRUE | 54.25440517 |
| rs35633876 | T | G | 0.481841 | -0.0344611 | 0.00406887 | 2.39994E-17 | TRUE | 71.73162068 |
| rs36018387 | T | C | 0.105092 | -0.0442699 | 0.00662522 | 6.4003E-12 | TRUE | 44.64948701 |
| rs4008004 | A | C | 0.221842 | 0.0309046 | 0.00489606 | 1.89998E-10 | TRUE | 39.84306969 |
| rs4299376 | T | G | 0.676279 | -0.0387395 | 0.00433864 | 2.49977E-19 | TRUE | 79.72614963 |
| rs4665972 | C | T | 0.604642 | -0.0513405 | 0.00416674 | 1.29987E-35 | TRUE | 151.8194397 |
| rs4704210 | C | G | 0.374212 | 0.0511244 | 0.00418591 | 1.99986E-35 | TRUE | 149.1683464 |
| rs4947302 | T | C | 0.064792 | 0.0459621 | 0.00824431 | 4.79999E-10 | TRUE | 31.08072204 |
| rs534417 | G | A | 0.875005 | 0.0359986 | 0.00610468 | 2.5E-09 | TRUE | 34.77326058 |
| rs55747707 | A | G | 0.203615 | -0.0449899 | 0.00504536 | 1.50003E-18 | TRUE | 79.51439084 |
| rs56322906 | A | G | 0.035157 | -0.0883014 | 0.0110068 | 2.90001E-15 | TRUE | 64.35955435 |
| rs58542926 | T | C | 0.074383 | -0.109822 | 0.00772171 | 7.50067E-48 | TRUE | 202.2794308 |
| rs602633 | G | T | 0.78295 | 0.0531806 | 0.00490259 | 7.50067E-28 | TRUE | 117.667172 |
| rs633695 | G | A | 0.292348 | 0.0697 | 0.00446965 | 1.99986E-54 | TRUE | 243.1747751 |
| rs6471717 | A | G | 0.663091 | -0.0308556 | 0.00429616 | 1.39991E-13 | TRUE | 51.58305948 |
| rs6602911 | T | C | 0.360079 | 0.0238048 | 0.00421825 | 1.40001E-08 | TRUE | 31.84670051 |
| rs6882345 | A | G | 0.632863 | 0.0441266 | 0.00419712 | 1.99986E-26 | TRUE | 110.5345784 |
| rs693 | A | G | 0.520883 | 0.0614441 | 0.00405281 | 3.40017E-54 | TRUE | 229.851788 |
| rs7139079 | A | G | 0.592501 | -0.0259545 | 0.00413291 | 4.79999E-10 | TRUE | 39.43786878 |
| rs740516 | G | C | 0.151132 | -0.03364 | 0.00568287 | 1.89998E-09 | TRUE | 35.04100323 |
| rs7707394 | A | G | 0.357106 | 0.0316688 | 0.00422065 | 3.90032E-14 | TRUE | 56.29949971 |
| rs7750288 | G | A | 0.285011 | 0.0265974 | 0.0044814 | 1.09999E-09 | TRUE | 35.22499578 |
| rs77960347 | G | A | 0.013239 | 0.248604 | 0.0176915 | 1.10002E-45 | TRUE | 197.4635364 |
| rs7816447 | C | T | 0.100848 | -0.0487086 | 0.00671655 | 8.19974E-13 | TRUE | 52.5918914 |
| rs79429216 | A | G | 0.012713 | 0.146817 | 0.0180405 | 1.59993E-16 | TRUE | 66.23012141 |
| rs9302635 | C | T | 0.182464 | -0.0283511 | 0.00523184 | 3.40001E-08 | TRUE | 29.36506184 |
| rs9304381 | T | C | 0.818434 | 0.0628471 | 0.00525856 | 1E-33 | TRUE | 142.8357166 |
| rs964184 | C | G | 0.867229 | -0.145606 | 0.00596821 | 1.9999E-136 | TRUE | 595.2101804 |

Abbreviation: SNP, single nucleotide polymorphism; SE, standard error; EAF, effect allele frequency;

Supplementary Table 6 Mendelian randomization analysis of type 2 diabetes and linoleic acid.

| SNP | effect_allele | other_allele | eaf | beta | se | pval | mr_keep | F(β2)/(se2) |
| --- | --- | --- | --- | --- | --- | --- | --- | --- |
| rs10077431 | A | C | 0.214668 | -0.0487 | 0.0089 | 4.75499E-08 | TRUE | 29.94180028 |
| rs10087241 | A | G | 0.594893 | -0.0475 | 0.008 | 2.79602E-09 | TRUE | 35.25390625 |
| rs10100265 | C | A | 0.61049 | -0.0491 | 0.0079 | 6.28796E-10 | TRUE | 38.62858516 |
| rs10114341 | C | T | 0.440754 | -0.0409 | 0.0072 | 1.15101E-08 | TRUE | 32.26871142 |
| rs10401969 | C | T | 0.0765858 | 0.0921 | 0.0133 | 4.13143E-12 | TRUE | 47.95302165 |
| rs1050226 | G | A | 0.406792 | -0.0491 | 0.0074 | 3.34195E-11 | TRUE | 44.02501826 |
| rs1061813 | A | G | 0.537119 | -0.0429 | 0.0073 | 3.37202E-09 | TRUE | 34.5357478 |
| rs1063355 | G | T | 0.602436 | 0.0709 | 0.0079 | 3.71535E-19 | TRUE | 80.54494472 |
| rs10740322 | A | G | 0.687104 | 0.0477 | 0.0085 | 2.10902E-08 | TRUE | 31.49190311 |
| rs10811661 | C | T | 0.173605 | -0.1569 | 0.0098 | 4.13238E-58 | TRUE | 256.3266347 |
| rs10830963 | G | C | 0.275768 | 0.0909 | 0.008 | 5.84655E-30 | TRUE | 129.1064063 |
| rs10842994 | T | C | 0.197025 | -0.0755 | 0.0091 | 1.01508E-16 | TRUE | 68.83528559 |
| rs10974438 | C | A | 0.351215 | 0.0591 | 0.0075 | 3.01301E-15 | TRUE | 62.0944 |
| rs11098676 | C | T | 0.787639 | 0.054 | 0.0096 | 2.02698E-08 | TRUE | 31.640625 |
| rs11107116 | T | G | 0.219714 | 0.0467 | 0.0085 | 3.74999E-08 | TRUE | 30.18532872 |
| rs1111875 | T | C | 0.408262 | -0.0948 | 0.0072 | 3.61493E-39 | TRUE | 173.3611111 |
| rs11257655 | T | C | 0.206773 | 0.0737 | 0.0087 | 1.96607E-17 | TRUE | 71.76231999 |
| rs1127655 | T | C | 0.529064 | -0.0438 | 0.0079 | 2.47098E-08 | TRUE | 30.7393046 |
| rs11708067 | G | A | 0.23899 | -0.0965 | 0.0086 | 5.93335E-29 | TRUE | 125.9092753 |
| rs11925227 | A | G | 0.183439 | -0.0534 | 0.0095 | 2.24999E-08 | TRUE | 31.59623269 |
| rs11926707 | C | T | 0.625556 | 0.0463 | 0.0082 | 1.68601E-08 | TRUE | 31.88117192 |
| rs12088739 | G | A | 0.0898481 | -0.0884 | 0.013 | 9.79264E-12 | TRUE | 46.24 |
| rs12299509 | G | A | 0.478622 | 0.0467 | 0.0073 | 2.08699E-10 | TRUE | 40.92493901 |
| rs12617659 | T | C | 0.147238 | -0.0685 | 0.0103 | 2.82683E-11 | TRUE | 44.22895655 |
| rs12910825 | G | A | 0.360391 | 0.0517 | 0.0074 | 2.16421E-12 | TRUE | 48.81099343 |
| rs12945601 | C | T | 0.613603 | -0.048 | 0.008 | 1.71799E-09 | TRUE | 36 |
| rs12970134 | A | G | 0.26512 | 0.0555 | 0.008 | 5.30884E-12 | TRUE | 48.12890625 |
| rs13239186 | T | C | 0.302029 | 0.0539 | 0.0085 | 2.70402E-10 | TRUE | 40.21051903 |
| rs13330951 | G | A | 0.488314 | -0.0456 | 0.0081 | 1.53801E-08 | TRUE | 31.69272977 |
| rs13389219 | T | C | 0.394368 | -0.0722 | 0.0074 | 2.1062E-22 | TRUE | 95.19430241 |
| rs1359790 | A | G | 0.2867 | -0.0796 | 0.008 | 2.79512E-23 | TRUE | 99.0025 |
| rs1496653 | G | A | 0.204782 | -0.0769 | 0.0088 | 2.57217E-18 | TRUE | 76.3637655 |
| rs1552224 | C | A | 0.15421 | -0.1034 | 0.0101 | 8.63575E-25 | TRUE | 104.8089403 |
| rs16988333 | G | A | 0.0904035 | -0.0745 | 0.013 | 9.16896E-09 | TRUE | 32.84171598 |
| rs17086692 | T | G | 0.313426 | -0.0467 | 0.0084 | 2.48102E-08 | TRUE | 30.90830499 |
| rs17168486 | T | C | 0.173603 | 0.0742 | 0.0094 | 2.17721E-15 | TRUE | 62.30918968 |
| rs17405722 | A | G | 0.0741526 | 0.087 | 0.0146 | 2.27798E-09 | TRUE | 35.50853819 |
| rs17411031 | G | C | 0.261729 | -0.045 | 0.0081 | 3.03501E-08 | TRUE | 30.86419753 |
| rs1758632 | G | C | 0.623407 | 0.0491 | 0.0081 | 1.36E-09 | TRUE | 36.74455114 |
| rs17631783 | T | C | 0.26346 | -0.0487 | 0.0089 | 3.94903E-08 | TRUE | 29.94180028 |
| rs17791513 | G | A | 0.0605985 | -0.1027 | 0.0148 | 4.61424E-12 | TRUE | 48.1523466 |
| rs1801214 | T | C | 0.599585 | 0.0903 | 0.0074 | 5.51569E-34 | TRUE | 148.9059533 |
| rs1899951 | T | C | 0.123288 | -0.1118 | 0.0109 | 1.63682E-24 | TRUE | 105.2036024 |
| rs2237892 | T | C | 0.0624896 | -0.096 | 0.0157 | 8.74601E-10 | TRUE | 37.38894073 |
| rs2246618 | T | C | 0.307253 | 0.0513 | 0.0084 | 1.20301E-09 | TRUE | 37.29719388 |
| rs2261181 | T | C | 0.09647 | 0.0985 | 0.0118 | 9.1791E-17 | TRUE | 69.68004884 |
| rs2294120 | G | A | 0.455879 | -0.0443 | 0.0079 | 1.61801E-08 | TRUE | 31.44512097 |
| rs2296173 | G | A | 0.212011 | 0.065 | 0.0087 | 7.65773E-14 | TRUE | 55.81979125 |
| rs2299383 | T | C | 0.423455 | 0.0412 | 0.0073 | 1.49001E-08 | TRUE | 31.85288047 |
| rs243019 | C | T | 0.455831 | 0.0566 | 0.0071 | 2.28981E-15 | TRUE | 63.55008927 |
| rs2493394 | G | A | 0.107338 | 0.073 | 0.0113 | 1.15101E-10 | TRUE | 41.73388676 |
| rs2796441 | A | G | 0.416458 | -0.0715 | 0.0073 | 1.962E-22 | TRUE | 95.93263276 |
| rs2820426 | G | A | 0.610058 | 0.0521 | 0.0073 | 1.30197E-12 | TRUE | 50.93657347 |
| rs2867125 | C | T | 0.827825 | 0.0601 | 0.0096 | 4.32504E-10 | TRUE | 39.19281684 |
| rs2908282 | A | G | 0.177395 | 0.0552 | 0.0094 | 4.25001E-09 | TRUE | 34.48438207 |
| rs2925979 | C | T | 0.70085 | -0.0534 | 0.0078 | 9.0615E-12 | TRUE | 46.86982249 |
| rs2943656 | G | A | 0.635133 | 0.0902 | 0.0074 | 6.6973E-34 | TRUE | 148.5763331 |
| rs3217992 | T | C | 0.369901 | 0.0527 | 0.0073 | 7.2277E-13 | TRUE | 52.11653218 |
| rs340874 | C | T | 0.563904 | 0.0626 | 0.0073 | 8.40621E-18 | TRUE | 73.5364984 |
| rs348330 | A | G | 0.633451 | -0.0487 | 0.0081 | 1.86402E-09 | TRUE | 36.14830056 |
| rs3756784 | G | T | 0.185838 | 0.0505 | 0.0091 | 2.58899E-08 | TRUE | 30.7964014 |
| rs3802177 | A | G | 0.311281 | -0.1217 | 0.008 | 2.32113E-52 | TRUE | 231.4201563 |
| rs459193 | G | A | 0.745338 | 0.0711 | 0.0083 | 8.80846E-18 | TRUE | 73.38089708 |
| rs4622883 | G | A | 0.508544 | -0.0435 | 0.0078 | 3.01599E-08 | TRUE | 31.10207101 |
| rs4686471 | C | T | 0.609775 | 0.0534 | 0.0081 | 4.28154E-11 | TRUE | 43.46227709 |
| rs4812829 | A | G | 0.160871 | 0.0532 | 0.0095 | 2.44298E-08 | TRUE | 31.36 |
| rs4823182 | G | A | 0.335748 | 0.0482 | 0.0077 | 3.35799E-10 | TRUE | 39.18434812 |
| rs4865796 | A | G | 0.69309 | 0.053 | 0.0078 | 1.32892E-11 | TRUE | 46.17028271 |
| rs516946 | C | T | 0.760633 | 0.0824 | 0.0085 | 3.15864E-22 | TRUE | 93.97591696 |
| rs5215 | T | C | 0.639941 | -0.0678 | 0.0073 | 2.08882E-20 | TRUE | 86.26083693 |
| rs55966194 | G | C | 0.281312 | -0.0526 | 0.0088 | 2.25398E-09 | TRUE | 35.72778926 |
| rs576674 | A | G | 0.832515 | -0.0654 | 0.0097 | 1.79184E-11 | TRUE | 45.45817834 |
| rs6059662 | G | A | 0.663176 | 0.0446 | 0.0079 | 1.51199E-08 | TRUE | 31.87245634 |
| rs61953351 | T | G | 0.249902 | -0.07 | 0.0091 | 1.97606E-14 | TRUE | 59.17159763 |
| rs622217 | C | T | 0.483932 | -0.0485 | 0.0077 | 3.12903E-10 | TRUE | 39.67363805 |
| rs6515236 | C | A | 0.24933 | -0.0504 | 0.0091 | 3.34303E-08 | TRUE | 30.67455621 |
| rs67232546 | T | C | 0.209222 | 0.0596 | 0.0096 | 4.66101E-10 | TRUE | 38.54340278 |
| rs6767484 | G | A | 0.312261 | 0.1209 | 0.0076 | 2.70085E-56 | TRUE | 253.061115 |
| rs6785040 | C | T | 0.15066 | -0.0633 | 0.0111 | 1.26401E-08 | TRUE | 32.52081812 |
| rs6795735 | T | C | 0.410912 | -0.0558 | 0.0073 | 1.63005E-14 | TRUE | 58.42822293 |
| rs6878122 | A | G | 0.681791 | -0.0564 | 0.0079 | 1.18796E-12 | TRUE | 50.96875501 |
| rs6960043 | C | T | 0.521837 | 0.064 | 0.0071 | 3.61077E-19 | TRUE | 81.2537195 |
| rs7144011 | T | G | 0.221063 | 0.0482 | 0.0085 | 1.63599E-08 | TRUE | 32.15557093 |
| rs7177055 | A | G | 0.718289 | 0.0647 | 0.0079 | 2.746E-16 | TRUE | 67.0740266 |
| rs7240767 | C | T | 0.383677 | 0.0451 | 0.0081 | 2.157E-08 | TRUE | 31.00152416 |
| rs72802358 | C | G | 0.101594 | -0.1168 | 0.0133 | 1.97015E-18 | TRUE | 77.12273164 |
| rs72892910 | T | G | 0.172394 | 0.0648 | 0.0099 | 6.42836E-11 | TRUE | 42.84297521 |
| rs735949 | C | T | 0.14115 | -0.0711 | 0.0106 | 1.94581E-11 | TRUE | 44.99118904 |
| rs753270 | C | T | 0.583535 | 0.0528 | 0.0079 | 2.70209E-11 | TRUE | 44.66976446 |
| rs7561798 | G | A | 0.482171 | 0.04 | 0.0072 | 2.79402E-08 | TRUE | 30.86419753 |
| rs7572970 | G | A | 0.722047 | 0.059 | 0.0087 | 1.39091E-11 | TRUE | 45.99022328 |
| rs7607777 | T | G | 0.105906 | -0.137 | 0.0125 | 9.39723E-28 | TRUE | 120.1216 |
| rs7674212 | T | G | 0.408864 | -0.0465 | 0.0075 | 6.18201E-10 | TRUE | 38.44 |
| rs7685296 | T | C | 0.279365 | -0.0511 | 0.0081 | 2.31702E-10 | TRUE | 39.79896357 |
| rs7729395 | T | C | 0.0509409 | 0.1373 | 0.016 | 1.10103E-17 | TRUE | 73.63785156 |
| rs7756992 | G | A | 0.266896 | 0.1297 | 0.0078 | 5.99929E-62 | TRUE | 276.4972058 |
| rs7786095 | G | A | 0.10386 | -0.0743 | 0.0129 | 9.64295E-09 | TRUE | 33.174028 |
| rs780094 | C | T | 0.612845 | 0.0692 | 0.0074 | 5.15941E-21 | TRUE | 87.4477721 |
| rs7845219 | C | T | 0.492786 | -0.0422 | 0.0072 | 4.54496E-09 | TRUE | 34.35262346 |
| rs7903146 | T | C | 0.291585 | 0.3059 | 0.0077 | 1E-200 | TRUE | 1578.256198 |
| rs7929543 | C | A | 0.0831599 | 0.0828 | 0.0138 | 2.19902E-09 | TRUE | 36 |
| rs7955901 | T | C | 0.556668 | -0.0444 | 0.0072 | 7.16094E-10 | TRUE | 38.02777778 |
| rs8068804 | A | G | 0.325097 | 0.0587 | 0.0078 | 4.41062E-14 | TRUE | 56.63527285 |
| rs8108269 | G | T | 0.281015 | 0.0644 | 0.0079 | 3.11387E-16 | TRUE | 66.45345297 |
| rs825476 | T | C | 0.580549 | 0.0524 | 0.0073 | 6.80456E-13 | TRUE | 51.52486395 |
| rs840967 | A | C | 0.605922 | -0.0497 | 0.008 | 5.44202E-10 | TRUE | 38.59515625 |
| rs849135 | A | G | 0.499052 | -0.0999 | 0.0072 | 1.04112E-43 | TRUE | 192.515625 |
| rs853974 | C | T | 0.737586 | -0.0601 | 0.0088 | 7.85778E-12 | TRUE | 46.64269112 |
| rs9369425 | A | G | 0.708185 | -0.0546 | 0.0085 | 1.12899E-10 | TRUE | 41.2617301 |
| rs963740 | T | A | 0.294299 | -0.0479 | 0.0086 | 2.23198E-08 | TRUE | 31.02230936 |
| rs9844972 | C | G | 0.0697229 | 0.0956 | 0.0148 | 1.02601E-10 | TRUE | 41.72461651 |
| rs9894220 | G | A | 0.43374 | -0.0585 | 0.0079 | 1.51705E-13 | TRUE | 54.83496235 |
| rs9928094 | G | A | 0.42602 | 0.1045 | 0.0072 | 3.58922E-47 | TRUE | 210.6529707 |
| rs993380 | G | A | 0.66555 | -0.0507 | 0.0081 | 4.58701E-10 | TRUE | 39.17832647 |
| rs9940149 | A | G | 0.178556 | -0.058 | 0.0095 | 9.29095E-10 | TRUE | 37.27423823 |

Abbreviation: SNP, single nucleotide polymorphism; SE, standard error; EAF, effect allele frequency;

Supplementary Table 7 Mendelian randomization analysis of fasting blood glucose and linoleic acid.

| SNP | effect_allele | other_allele | eaf | beta | se | pval | mr_keep | F(β2)/(se2) |
| --- | --- | --- | --- | --- | --- | --- | --- | --- |
| rs10305457 | T | C | 0.065 | 0.0235 | 0.0032 | 1.20893E-14 | TRUE | 53.93066406 |
| rs1057394 | A | G | 0.625 | -0.0124 | 0.0018 | 1.91117E-12 | TRUE | 47.45679012 |
| rs10811660 | A | G | 0.165 | -0.0223 | 0.0022 | 7.9378E-25 | TRUE | 102.7458678 |
| rs10830963 | G | C | 0.286 | 0.0772 | 0.0019 | 1E-200 | TRUE | 1650.925208 |
| rs10838524 | G | A | 0.52 | -0.0238 | 0.0016 | 1.55883E-40 | TRUE | 221.265625 |
| rs10838693 | C | G | 0.314 | 0.0177 | 0.0018 | 3.44191E-23 | TRUE | 96.69444444 |
| rs10974438 | C | A | 0.38 | 0.0198 | 0.0017 | 9.84691E-31 | TRUE | 135.6539792 |
| rs11603349 | C | T | 0.168 | -0.0236 | 0.0022 | 3.11602E-25 | TRUE | 115.0743802 |
| rs11610045 | A | G | 0.454 | 0.0144 | 0.0019 | 3.25987E-13 | TRUE | 57.44044321 |
| rs11619319 | G | A | 0.234 | 0.0173 | 0.002 | 3.41193E-20 | TRUE | 74.8225 |
| rs11708067 | G | A | 0.177 | -0.0281 | 0.002 | 1.62592E-43 | TRUE | 197.4025 |
| rs12055786 | T | C | 0.384 | 0.012 | 0.0017 | 1.166E-11 | TRUE | 49.82698962 |
| rs12541643 | T | C | 0.479 | 0.0118 | 0.0019 | 4.51097E-09 | TRUE | 38.57063712 |
| rs1260326 | C | T | 0.587 | 0.0282 | 0.0017 | 4.48126E-65 | TRUE | 275.1695502 |
| rs12784552 | G | A | 0.076 | -0.0329 | 0.003 | 2.86286E-31 | TRUE | 120.2677778 |
| rs12888855 | A | C | 0.189 | -0.0135 | 0.002 | 6.01866E-12 | TRUE | 45.5625 |
| rs12898997 | T | C | 0.598 | -0.0098 | 0.0017 | 4.64098E-09 | TRUE | 33.23183391 |
| rs157512 | C | T | 0.27 | -0.0134 | 0.0021 | 5.426E-10 | TRUE | 40.71655329 |
| rs1604038 | T | C | 0.288 | -0.0198 | 0.0018 | 4.46786E-28 | TRUE | 121 |
| rs16851397 | G | A | 0.045 | -0.0327 | 0.0042 | 1.26009E-12 | TRUE | 60.61734694 |
| rs16913693 | G | T | 0.029 | -0.0394 | 0.0049 | 2.81968E-16 | TRUE | 64.6547272 |
| rs17168486 | T | C | 0.176 | 0.028 | 0.0021 | 4.16965E-36 | TRUE | 177.7777778 |
| rs17265513 | C | T | 0.204 | 0.0158 | 0.0021 | 5.10152E-14 | TRUE | 56.60770975 |
| rs17270243 | G | A | 0.24 | 0.0104 | 0.0021 | 3.61601E-08 | TRUE | 24.5260771 |
| rs17437560 | T | C | 0.11 | -0.0175 | 0.0032 | 3.33096E-08 | TRUE | 29.90722656 |
| rs174583 | T | C | 0.375 | -0.0168 | 0.0017 | 3.37132E-22 | TRUE | 97.66089965 |
| rs1820176 | C | T | 0.296 | -0.0247 | 0.002 | 1.90502E-34 | TRUE | 152.5225 |
| rs189548 | A | G | 0.723 | -0.0123 | 0.002 | 2.81099E-09 | TRUE | 37.8225 |
| rs194518 | A | G | 0.52 | 0.0102 | 0.0018 | 8.75709E-09 | TRUE | 32.11111111 |
| rs2075423 | T | G | 0.376 | -0.0161 | 0.0017 | 3.18273E-21 | TRUE | 89.69204152 |
| rs2238435 | G | C | 0.619 | -0.0112 | 0.0019 | 3.82402E-09 | TRUE | 34.74792244 |
| rs2461385 | C | T | 0.852 | -0.0217 | 0.0024 | 2.73023E-19 | TRUE | 81.75173611 |
| rs2595701 | G | A | 0.683 | -0.0189 | 0.0021 | 4.47816E-19 | TRUE | 81 |
| rs2657879 | G | A | 0.202 | 0.0119 | 0.0022 | 7.33398E-09 | TRUE | 29.25826446 |
| rs2839671 | A | G | 0.164 | -0.016 | 0.0022 | 8.37915E-14 | TRUE | 52.89256198 |
| rs348330 | A | G | 0.631 | -0.0122 | 0.002 | 3.03599E-10 | TRUE | 37.21 |
| rs35889227 | T | G | 0.617 | -0.013 | 0.0019 | 3.36504E-10 | TRUE | 46.81440443 |
| rs3778321 | A | G | 0.176 | -0.0186 | 0.0021 | 3.15718E-17 | TRUE | 78.44897959 |
| rs3829109 | A | G | 0.276 | -0.0163 | 0.002 | 1.08693E-15 | TRUE | 66.4225 |
| rs3842753 | G | T | 0.72 | -0.0134 | 0.0022 | 2.84099E-09 | TRUE | 37.09917355 |
| rs39713 | T | C | 0.062 | -0.0169 | 0.0031 | 1.76701E-08 | TRUE | 29.72008325 |
| rs4760278 | A | C | 0.181 | -0.011 | 0.002 | 3.33096E-08 | TRUE | 30.25 |
| rs4862423 | T | C | 0.401 | 0.0123 | 0.0019 | 4.44703E-10 | TRUE | 41.90858726 |
| rs507666 | A | G | 0.189 | 0.0164 | 0.0021 | 6.99198E-17 | TRUE | 60.98866213 |
| rs537183 | T | C | 0.64 | 0.0663 | 0.0017 | 1E-200 | TRUE | 1521 |
| rs58925536 | T | C | 0.032 | 0.0306 | 0.0053 | 5.82103E-09 | TRUE | 33.33428266 |
| rs6113722 | A | G | 0.066 | -0.0424 | 0.0044 | 7.65949E-25 | TRUE | 92.85950413 |
| rs6489811 | G | A | 0.512 | 0.011 | 0.0018 | 3.26701E-09 | TRUE | 37.34567901 |
| rs6538804 | G | C | 0.377 | -0.0142 | 0.0019 | 9.41239E-14 | TRUE | 55.85595568 |
| rs6598541 | G | A | 0.648 | -0.0114 | 0.0017 | 4.12382E-12 | TRUE | 44.96885813 |
| rs6662924 | A | C | 0.198 | 0.0143 | 0.0023 | 3.34103E-10 | TRUE | 38.65595463 |
| rs6808574 | C | T | 0.606 | 0.0127 | 0.0017 | 7.20941E-14 | TRUE | 55.80968858 |
| rs7012637 | A | G | 0.47 | -0.018 | 0.0017 | 9.74541E-25 | TRUE | 112.1107266 |
| rs7095788 | T | C | 0.358 | -0.0106 | 0.0018 | 1.97601E-09 | TRUE | 34.67901235 |
| rs7163757 | T | C | 0.433 | -0.0217 | 0.0016 | 2.64119E-36 | TRUE | 183.9414063 |
| rs7178572 | G | A | 0.679 | 0.0121 | 0.0018 | 7.08598E-10 | TRUE | 45.1882716 |
| rs7708285 | A | G | 0.686 | -0.0133 | 0.0019 | 1.253E-09 | TRUE | 49 |
| rs77981966 | T | C | 0.055 | -0.0246 | 0.0035 | 1.58016E-14 | TRUE | 49.40081633 |
| rs78132593 | A | C | 0.203 | -0.0147 | 0.0022 | 2.59801E-10 | TRUE | 44.64669421 |
| rs7903146 | T | C | 0.307 | 0.0259 | 0.0019 | 1.9948E-35 | TRUE | 185.8199446 |
| rs878521 | A | G | 0.249 | 0.0549 | 0.002 | 2.6485E-174 | TRUE | 753.5025 |
| rs896854 | C | T | 0.542 | -0.0099 | 0.0016 | 5.61203E-09 | TRUE | 38.28515625 |
| rs9348441 | A | T | 0.272 | 0.0176 | 0.0018 | 4.40251E-20 | TRUE | 95.60493827 |
| rs9650069 | T | C | 0.284 | -0.0286 | 0.0018 | 8.30615E-58 | TRUE | 252.4567901 |

Abbreviation: SNP, single nucleotide polymorphism; SE, standard error; EAF, effect allele frequency;

Supplementary Table 8 Mendelian randomization analysis of fasting insulin and linoleic acid.

| SNP | effect_allele | other_allele | eaf | beta | se | pval | mr_keep | F(β2)/(se2) |
| --- | --- | --- | --- | --- | --- | --- | --- | --- |
| rs10050393 | C | T | 0.46 | -0.009 | 0.0019 | 4.84295E-08 | TRUE | 22.43767313 |
| rs10865959 | C | G | 0.3 | 0.0138 | 0.0022 | 1.992E-08 | TRUE | 39.34710744 |
| rs116141873 | T | G | 0.032 | 0.0428 | 0.0059 | 1.42298E-11 | TRUE | 52.62395863 |
| rs11708067 | G | A | 0.177 | 0.0135 | 0.0023 | 1.299E-09 | TRUE | 34.45179584 |
| rs11727676 | C | T | 0.084 | 0.0203 | 0.0039 | 2.89801E-08 | TRUE | 27.09335963 |
| rs118164457 | C | T | 0.037 | 0.0345 | 0.0057 | 3.85798E-10 | TRUE | 36.63434903 |
| rs1206760 | A | G | 0.522 | -0.0112 | 0.0019 | 8.82104E-10 | TRUE | 34.74792244 |
| rs12454712 | C | T | 0.398 | -0.0142 | 0.0025 | 1.77701E-09 | TRUE | 32.2624 |
| rs1260326 | C | T | 0.587 | 0.0231 | 0.0019 | 8.41783E-38 | TRUE | 147.8144044 |
| rs13258890 | C | T | 0.252 | -0.0128 | 0.0025 | 2.76503E-08 | TRUE | 26.2144 |
| rs13389219 | T | C | 0.409 | -0.0199 | 0.0019 | 5.83848E-28 | TRUE | 109.6980609 |
| rs1351394 | C | T | 0.529 | 0.0111 | 0.0018 | 2.70801E-09 | TRUE | 38.02777778 |
| rs1474696 | G | A | 0.476 | 0.0147 | 0.0018 | 3.01926E-16 | TRUE | 66.69444444 |
| rs17036126 | T | C | 0.129 | 0.0209 | 0.003 | 1.279E-10 | TRUE | 48.53444444 |
| rs17331151 | T | C | 0.106 | -0.0162 | 0.0031 | 1.52398E-08 | TRUE | 27.30905307 |
| rs2108349 | A | G | 0.686 | -0.0115 | 0.002 | 1.133E-08 | TRUE | 33.0625 |
| rs2780215 | G | A | 0.042 | -0.0392 | 0.0063 | 1.064E-09 | TRUE | 38.71604938 |
| rs2845885 | T | C | 0.931 | -0.0204 | 0.0039 | 1.17999E-08 | TRUE | 27.36094675 |
| rs2943646 | G | A | 0.623 | 0.025 | 0.0019 | 8.47227E-39 | TRUE | 173.1301939 |
| rs35000407 | G | T | 0.117 | -0.0258 | 0.0028 | 1.50383E-21 | TRUE | 84.90306122 |
| rs3775380 | G | A | 0.5 | 0.0119 | 0.0018 | 1.47707E-11 | TRUE | 43.70679012 |
| rs459193 | G | A | 0.715 | 0.0181 | 0.0021 | 1.12305E-18 | TRUE | 74.28798186 |
| rs4865796 | A | G | 0.707 | 0.0165 | 0.002 | 7.32825E-17 | TRUE | 68.0625 |
| rs5017305 | T | A | 0.764 | -0.0137 | 0.0026 | 1.06699E-08 | TRUE | 27.7647929 |
| rs62271373 | A | T | 0.059 | 0.0256 | 0.0048 | 1.59599E-08 | TRUE | 28.44444444 |
| rs6487237 | A | C | 0.79 | 0.0154 | 0.0026 | 4.68198E-09 | TRUE | 35.08284024 |
| rs6674544 | A | G | 0.574 | 0.0177 | 0.002 | 6.97268E-21 | TRUE | 78.3225 |
| rs6855363 | C | T | 0.347 | -0.0125 | 0.002 | 4.03896E-08 | TRUE | 39.0625 |
| rs6905288 | A | G | 0.602 | 0.0112 | 0.0019 | 7.74997E-09 | TRUE | 34.74792244 |
| rs7012814 | A | G | 0.471 | -0.0219 | 0.0019 | 8.34257E-30 | TRUE | 132.8559557 |
| rs7133378 | A | G | 0.339 | -0.0127 | 0.002 | 5.99653E-11 | TRUE | 40.3225 |
| rs73013411 | A | C | 0.12 | -0.018 | 0.0032 | 2.08401E-08 | TRUE | 31.640625 |
| rs731839 | A | G | 0.658 | -0.0121 | 0.0019 | 3.86456E-11 | TRUE | 40.5567867 |
| rs75179845 | C | T | 0.079 | 0.0216 | 0.0035 | 6.05062E-11 | TRUE | 38.08653061 |
| rs7903146 | T | C | 0.307 | -0.0116 | 0.0021 | 1.237E-09 | TRUE | 30.51247166 |
| rs860598 | A | G | 0.824 | 0.0177 | 0.0025 | 6.8786E-12 | TRUE | 50.1264 |
| rs972283 | G | A | 0.544 | 0.0105 | 0.0019 | 1.093E-08 | TRUE | 30.5401662 |
| rs9884482 | C | T | 0.392 | 0.0125 | 0.0019 | 2.87872E-11 | TRUE | 43.28254848 |

Abbreviation: SNP, single nucleotide polymorphism; SE, standard error; EAF, effect allele frequency;

Supplementary Table 9 Mendelian randomization analysis of glycosylated hemoglobin and linoleic acid.

| SNP | effect_allele | other_allele | eaf | beta | se | pval | mr_keep | F(β2)/(se2) |
| --- | --- | --- | --- | --- | --- | --- | --- | --- |
| rs10151436 | T | A | 0.11 | -0.013 | 0.0021 | 3.85123E-11 | TRUE | 38.32199546 |
| rs10169706 | T | C | 0.04 | 0.026 | 0.0046 | 1.48201E-08 | TRUE | 31.94706994 |
| rs10405535 | G | A | 0.71 | -0.0122 | 0.0016 | 6.47441E-14 | TRUE | 58.140625 |
| rs10774624 | A | G | 0.525 | 0.0093 | 0.0013 | 4.16965E-14 | TRUE | 51.17751479 |
| rs10811661 | C | T | 0.165 | -0.0128 | 0.0017 | 1.735E-14 | TRUE | 56.69204152 |
| rs10830963 | G | C | 0.286 | 0.0197 | 0.0015 | 1.54099E-36 | TRUE | 172.4844444 |
| rs10946402 | G | T | 0.169 | 0.0101 | 0.0016 | 1.119E-10 | TRUE | 39.84765625 |
| rs11039154 | T | C | 0.277 | -0.0087 | 0.0014 | 3.10499E-09 | TRUE | 38.61734694 |
| rs11248914 | C | T | 0.302 | -0.0114 | 0.0014 | 1.42004E-14 | TRUE | 66.30612245 |
| rs11257655 | T | C | 0.241 | 0.011 | 0.0016 | 1.91117E-13 | TRUE | 47.265625 |
| rs11558471 | G | A | 0.293 | -0.0151 | 0.0014 | 3.38065E-25 | TRUE | 116.3316327 |
| rs11643024 | G | A | 0.697 | -0.0084 | 0.0015 | 7.97499E-10 | TRUE | 31.36 |
| rs11720108 | T | C | 0.182 | -0.0129 | 0.0015 | 2.95121E-18 | TRUE | 73.96 |
| rs117233107 | A | G | 0.02 | -0.047 | 0.0072 | 8.45279E-11 | TRUE | 42.61188272 |
| rs1175549 | C | A | 0.214 | -0.0098 | 0.0015 | 7.12689E-13 | TRUE | 42.68444444 |
| rs12491937 | G | A | 0.445 | -0.009 | 0.0013 | 1.4171E-13 | TRUE | 47.92899408 |
| rs12612492 | T | C | 0.148 | 0.0188 | 0.0019 | 1.88322E-26 | TRUE | 97.90581717 |
| rs1278769 | G | A | 0.769 | 0.0091 | 0.0015 | 5.51442E-12 | TRUE | 36.80444444 |
| rs13089972 | A | T | 0.584 | 0.0111 | 0.0014 | 1.87413E-15 | TRUE | 62.8622449 |
| rs13134327 | A | G | 0.331 | 0.0144 | 0.0014 | 2.80802E-26 | TRUE | 105.7959184 |
| rs13234131 | G | A | 0.124 | 0.0113 | 0.002 | 2.06101E-09 | TRUE | 31.9225 |
| rs13389076 | A | G | 0.034 | 0.0332 | 0.0038 | 3.03599E-18 | TRUE | 76.33240997 |
| rs13419763 | T | C | 0.588 | 0.008 | 0.0014 | 5.481E-09 | TRUE | 32.65306122 |
| rs1367173 | T | C | 0.106 | -0.0152 | 0.002 | 1.65997E-14 | TRUE | 57.76 |
| rs151165 | A | T | 0.397 | 0.0079 | 0.0014 | 2.037E-09 | TRUE | 31.84183673 |
| rs1535464 | A | G | 0.212 | -0.0086 | 0.0017 | 1.11099E-08 | TRUE | 25.5916955 |
| rs174559 | A | G | 0.285 | -0.0106 | 0.0014 | 3.31131E-13 | TRUE | 57.32653061 |
| rs17476364 | C | T | 0.099 | -0.0858 | 0.0023 | 1E-200 | TRUE | 1391.614367 |
| rs17533945 | C | T | 0.418 | 0.0128 | 0.0014 | 1.61696E-23 | TRUE | 83.59183673 |
| rs1800562 | A | G | 0.046 | -0.0383 | 0.0027 | 2.32702E-50 | TRUE | 201.2194787 |
| rs2015803 | T | C | 0.265 | 0.0112 | 0.0016 | 2.2751E-12 | TRUE | 49 |
| rs204995 | G | A | 0.219 | 0.0098 | 0.0018 | 1.93201E-09 | TRUE | 29.64197531 |
| rs2375278 | G | A | 0.824 | -0.0112 | 0.0017 | 1.04906E-11 | TRUE | 43.40484429 |
| rs2613522 | G | A | 0.26 | 0.027 | 0.0021 | 9.34329E-39 | TRUE | 165.3061224 |
| rs267738 | G | T | 0.203 | -0.0109 | 0.0016 | 1.14104E-11 | TRUE | 46.41015625 |
| rs2908277 | A | G | 0.117 | 0.0166 | 0.002 | 1.29003E-18 | TRUE | 68.89 |
| rs2971670 | T | C | 0.181 | 0.0316 | 0.0017 | 5.10035E-88 | TRUE | 345.5224913 |
| rs340882 | G | C | 0.58 | 0.0084 | 0.0013 | 1.483E-10 | TRUE | 41.75147929 |
| rs360147 | T | C | 0.264 | -0.0086 | 0.0015 | 2.07601E-09 | TRUE | 32.87111111 |
| rs3778321 | A | G | 0.176 | -0.0106 | 0.0016 | 4.18023E-11 | TRUE | 43.890625 |
| rs3829109 | A | G | 0.276 | -0.0086 | 0.0015 | 2.683E-08 | TRUE | 32.87111111 |
| rs452306 | T | C | 0.627 | -0.0098 | 0.0014 | 5.50554E-13 | TRUE | 49 |
| rs4727979 | C | A | 0.094 | -0.0121 | 0.0024 | 4.605E-08 | TRUE | 25.41840278 |
| rs4737009 | A | G | 0.262 | 0.0228 | 0.0015 | 8.29278E-56 | TRUE | 231.04 |
| rs4760682 | A | C | 0.817 | 0.0164 | 0.0018 | 3.19669E-20 | TRUE | 83.01234568 |
| rs4980325 | T | G | 0.532 | 0.0108 | 0.0014 | 4.70327E-14 | TRUE | 59.51020408 |
| rs560887 | C | T | 0.694 | 0.0307 | 0.0014 | 5.5463E-122 | TRUE | 480.8622449 |
| rs608793 | T | C | 0.479 | 0.0065 | 0.0013 | 4.54496E-08 | TRUE | 25 |
| rs61750929 | T | C | 0.041 | -0.0284 | 0.0029 | 9.48637E-24 | TRUE | 95.90487515 |
| rs6474359 | C | T | 0.022 | -0.0427 | 0.0038 | 1.91293E-33 | TRUE | 126.2666205 |
| rs651007 | T | C | 0.215 | 0.0108 | 0.0015 | 3.2802E-15 | TRUE | 51.84 |
| rs6798941 | T | C | 0.322 | 0.0086 | 0.0015 | 1.49101E-08 | TRUE | 32.87111111 |
| rs6804915 | A | C | 0.288 | -0.0108 | 0.0014 | 2.75931E-16 | TRUE | 59.51020408 |
| rs6877043 | C | T | 0.362 | -0.0085 | 0.0014 | 1.98701E-10 | TRUE | 36.8622449 |
| rs7042939 | G | A | 0.582 | -0.0102 | 0.0013 | 1.49692E-15 | TRUE | 61.56213018 |
| rs7127313 | T | C | 0.336 | 0.0066 | 0.0013 | 4.84797E-08 | TRUE | 25.77514793 |
| rs7190771 | A | G | 0.332 | 0.0085 | 0.0013 | 6.02143E-11 | TRUE | 42.75147929 |
| rs7198799 | T | C | 0.281 | 0.0083 | 0.0014 | 4.75905E-09 | TRUE | 35.14795918 |
| rs737092 | C | T | 0.499 | 0.0073 | 0.0013 | 7.56903E-09 | TRUE | 31.53254438 |
| rs7547793 | C | A | 0.88 | 0.0118 | 0.0021 | 6.61104E-09 | TRUE | 31.57369615 |
| rs76533333 | G | A | 0.087 | 0.0265 | 0.0025 | 2.80996E-29 | TRUE | 112.36 |
| rs7861647 | T | C | 0.193 | 0.0128 | 0.0016 | 4.49883E-14 | TRUE | 64 |
| rs7903146 | T | C | 0.307 | 0.0133 | 0.0014 | 1.044E-22 | TRUE | 90.25 |
| rs79403657 | G | C | 0.177 | 0.009 | 0.0017 | 2.03301E-08 | TRUE | 28.02768166 |
| rs8138197 | A | G | 0.488 | -0.0073 | 0.0014 | 3.53802E-08 | TRUE | 27.18877551 |
| rs837763 | T | C | 0.578 | 0.0176 | 0.0013 | 5.19757E-38 | TRUE | 183.2899408 |
| rs855791 | G | A | 0.6 | -0.0188 | 0.0013 | 1.3369E-56 | TRUE | 209.1360947 |
| rs857725 | G | T | 0.277 | 0.0208 | 0.0014 | 5.42625E-55 | TRUE | 220.7346939 |
| rs9376090 | C | T | 0.272 | -0.0247 | 0.0014 | 1.89714E-62 | TRUE | 311.2704082 |
| rs9818758 | A | G | 0.204 | 0.0131 | 0.0017 | 1.49417E-13 | TRUE | 59.38062284 |
| rs9909940 | T | C | 0.323 | 0.0322 | 0.0014 | 1.4289E-116 | TRUE | 529 |
| rs9914988 | A | G | 0.802 | 0.0125 | 0.0016 | 4.65586E-17 | TRUE | 61.03515625 |

Abbreviation: SNP, single nucleotide polymorphism; SE, standard error; EAF, effect allele frequency;

## Supplementary Figures


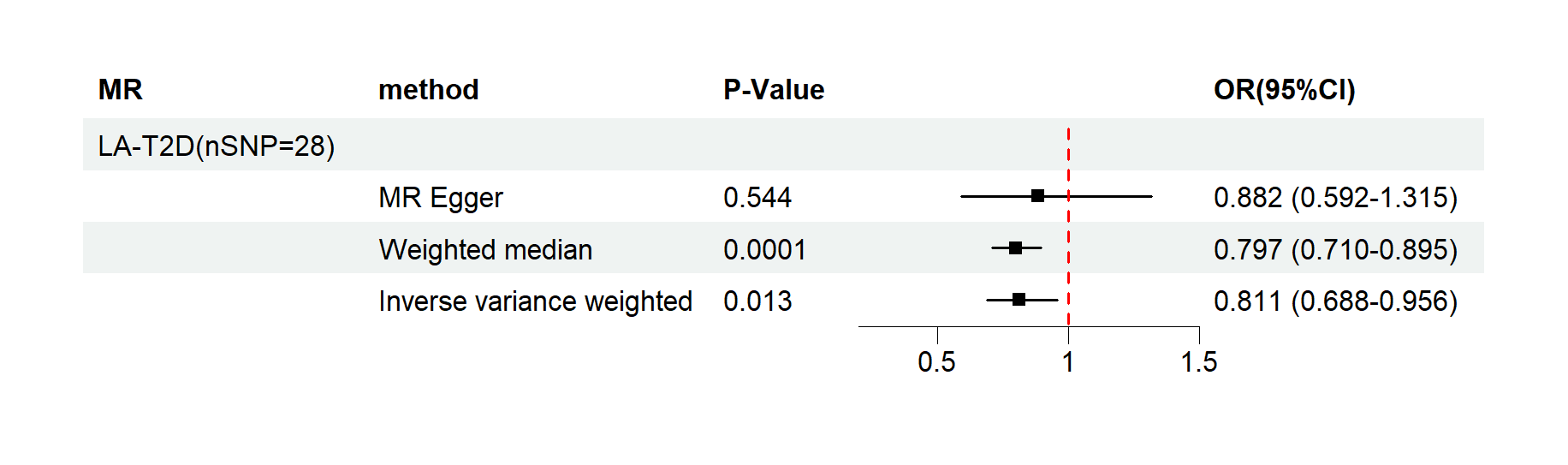


Supplementary Figure 1 Forest plot: Mendelian randomization analysis of linoleic acid and type 2 diabetes.


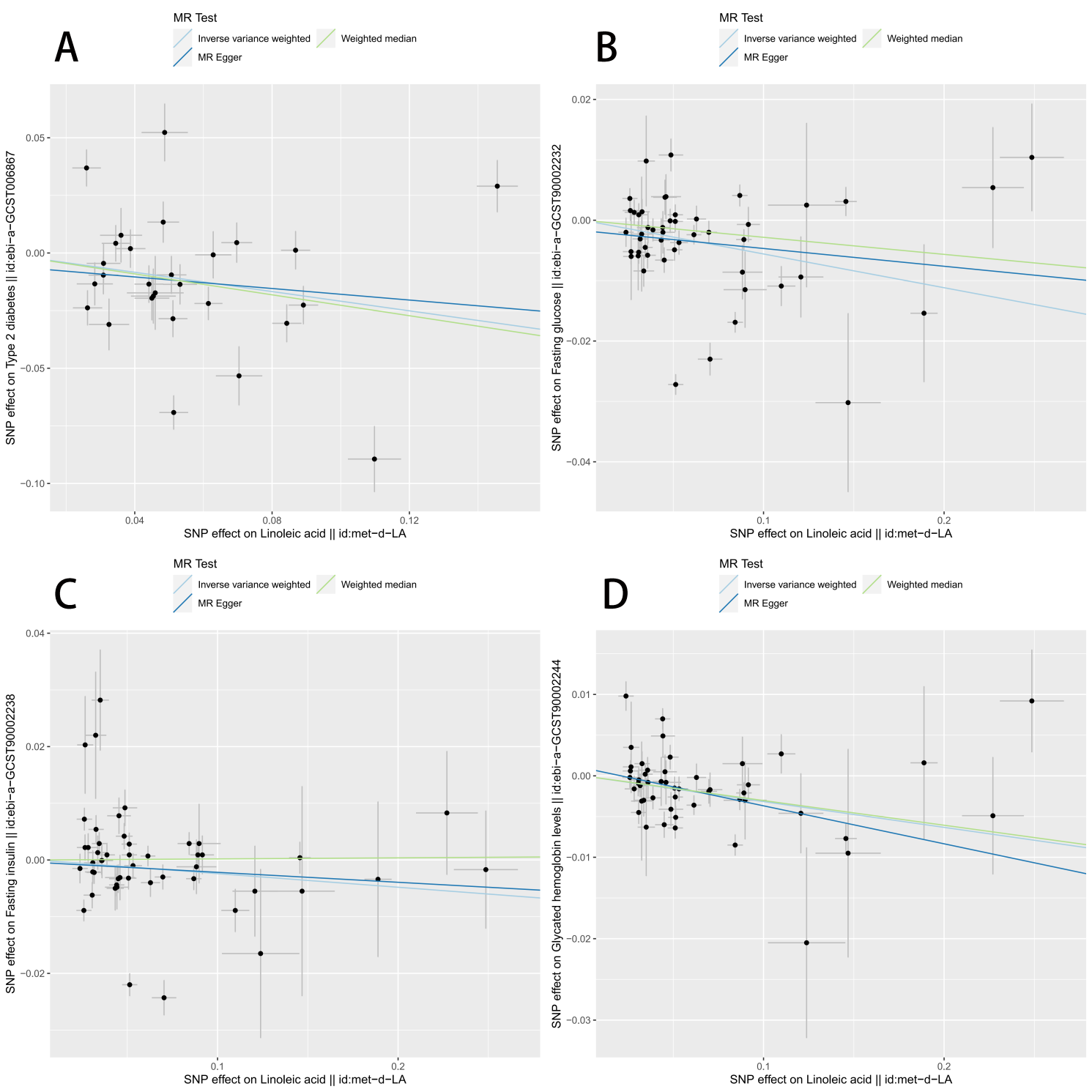


Supplementary Figure 2 Scatter plot: (A) LA and T2D; (B) LA and FBG; (C) LA and FI; (D) LA and HbA1c

Note: Each point on the scatter plot represents a SNP locus. The x-axis indicates the effect (β value) of the SNP on the exposure variable, while the y-axis indicates the effect (β value) of the SNP on the outcome variable. The colored line represents the MR fitting results.


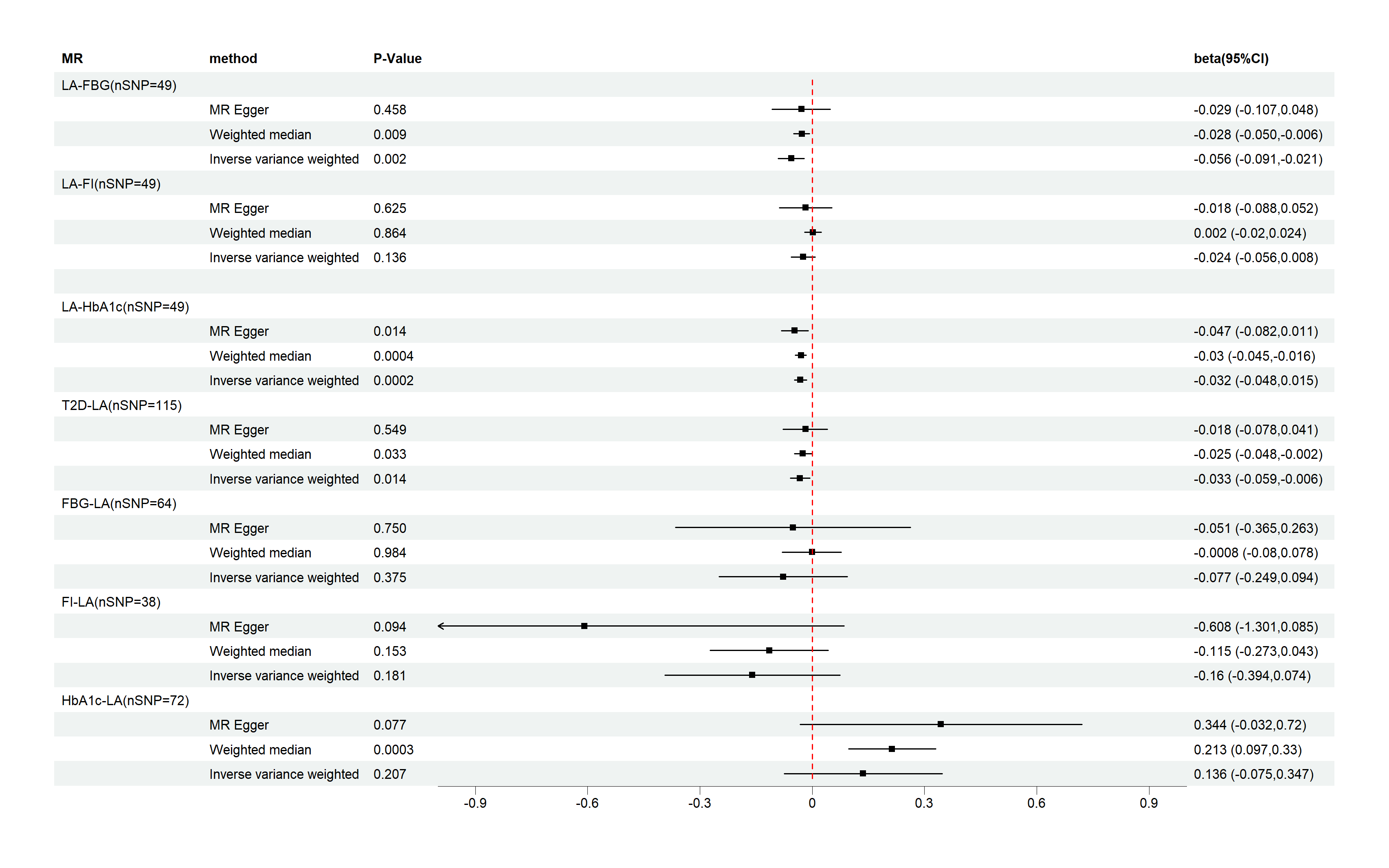


Supplementary Figure 3 Forest plot: LA and FBG; LA and FI; LA and HbA1c(A) ；T2D and LA; FBG and LA; FBG and LA; FBG and LA


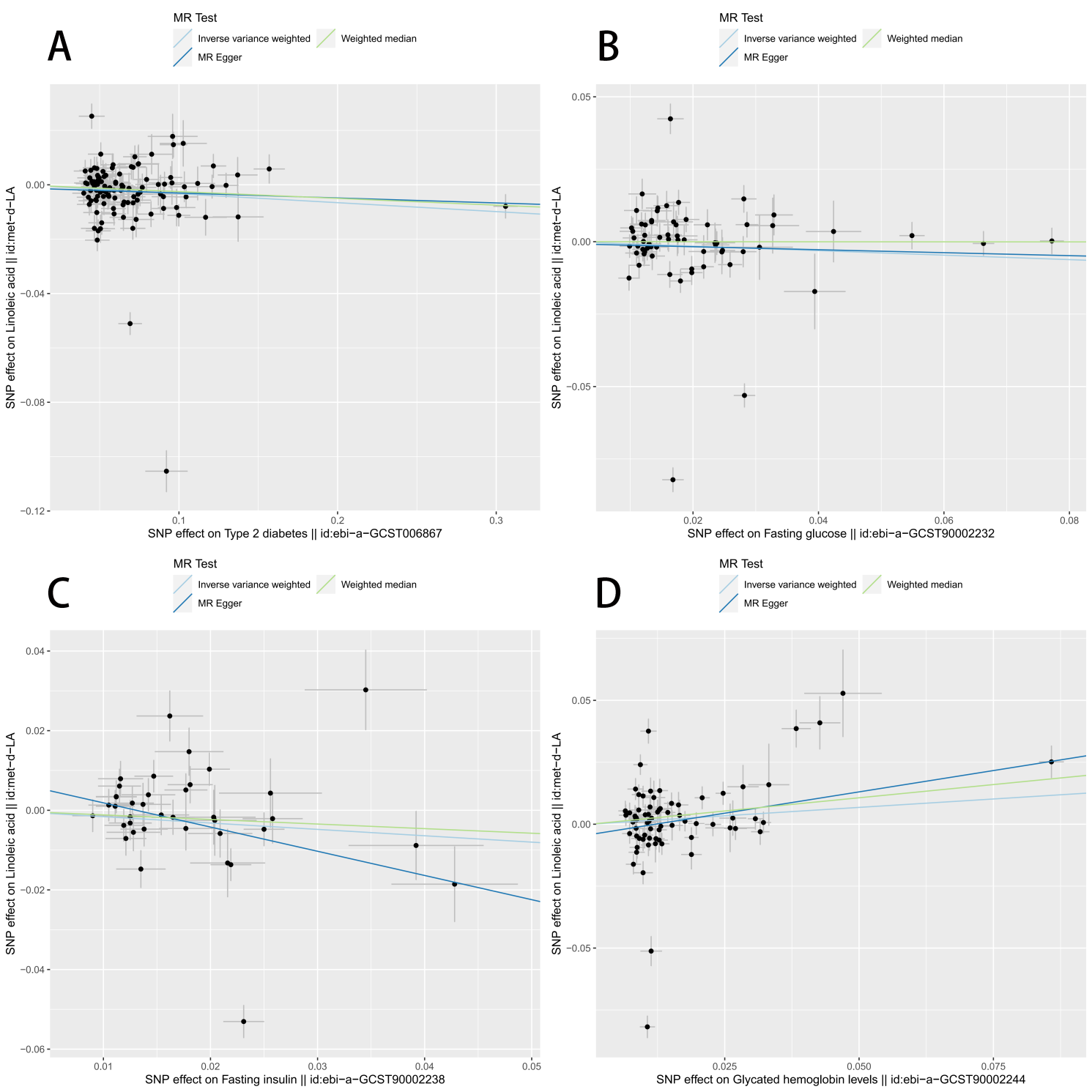


Supplementary Figure 4 Scatter plot: (A) T2D and LA; (B) FBG and LA; (C) FI and LA; (D) HbA1c and LA

Note: Each point on the scatter plot represents a SNP locus. The x-axis indicates the effect (β value) of the SNP on the exposure variable, while the y-axis indicates the effect (β value) of the SNP on the outcome variable. The colored line represents the MR fitting results.


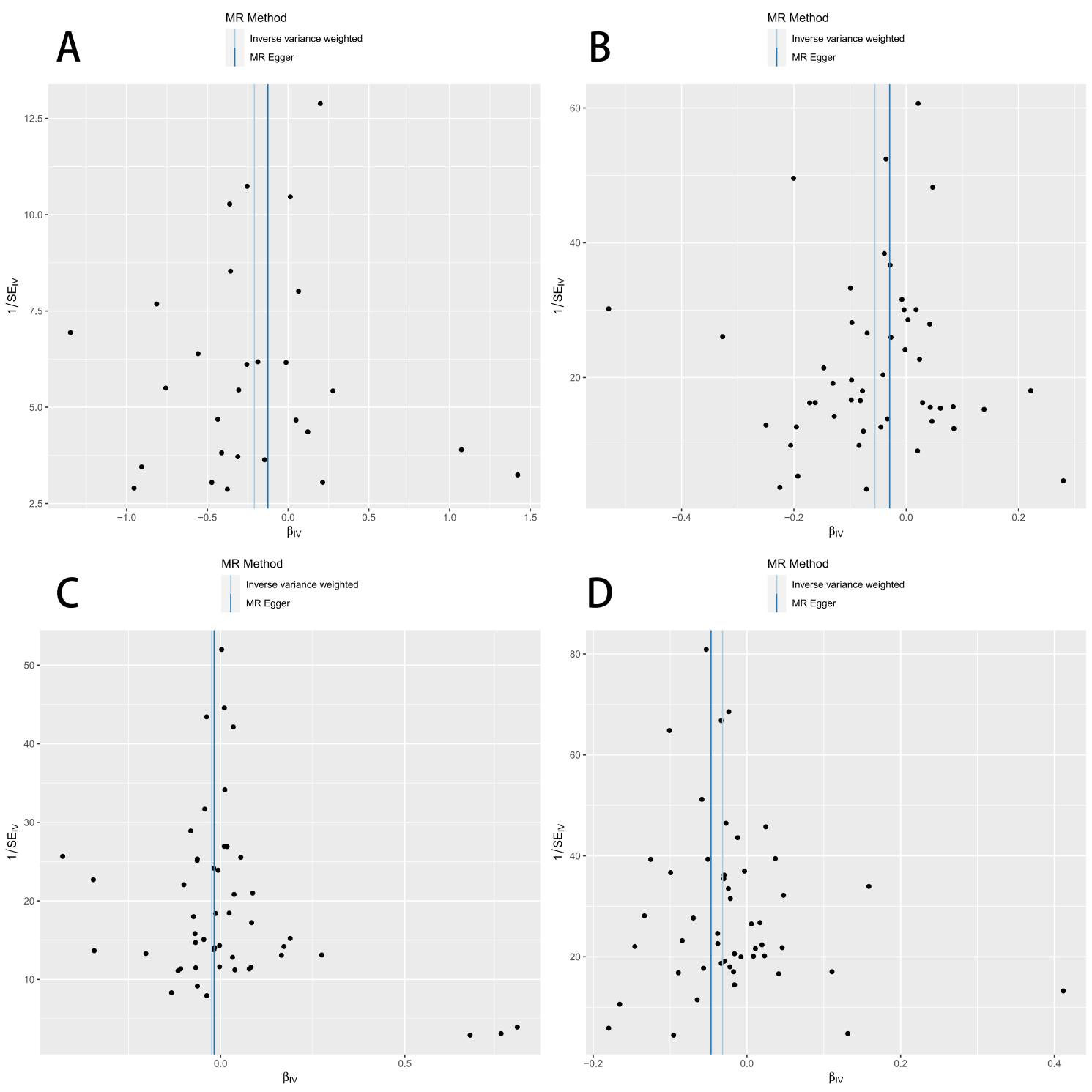


Supplementary Figure 5 Funnel plot: (A) LA and T2D; (B) LA and FBG; (C) LA and FI; (D) LA and HbA1c

Note: Each point on the scatter plot represents an instrumental variable. The x-axis shows the effect size (β value) from MR analysis based on individual SNPs. The y-axis represents the inverse of the standard error, indicating the weight of the SNP. A β value greater than 0 suggests that an increase in the exposure variable will result in an increase in the outcome variable. The colored line depicts the fitted effects from the corresponding MR method.


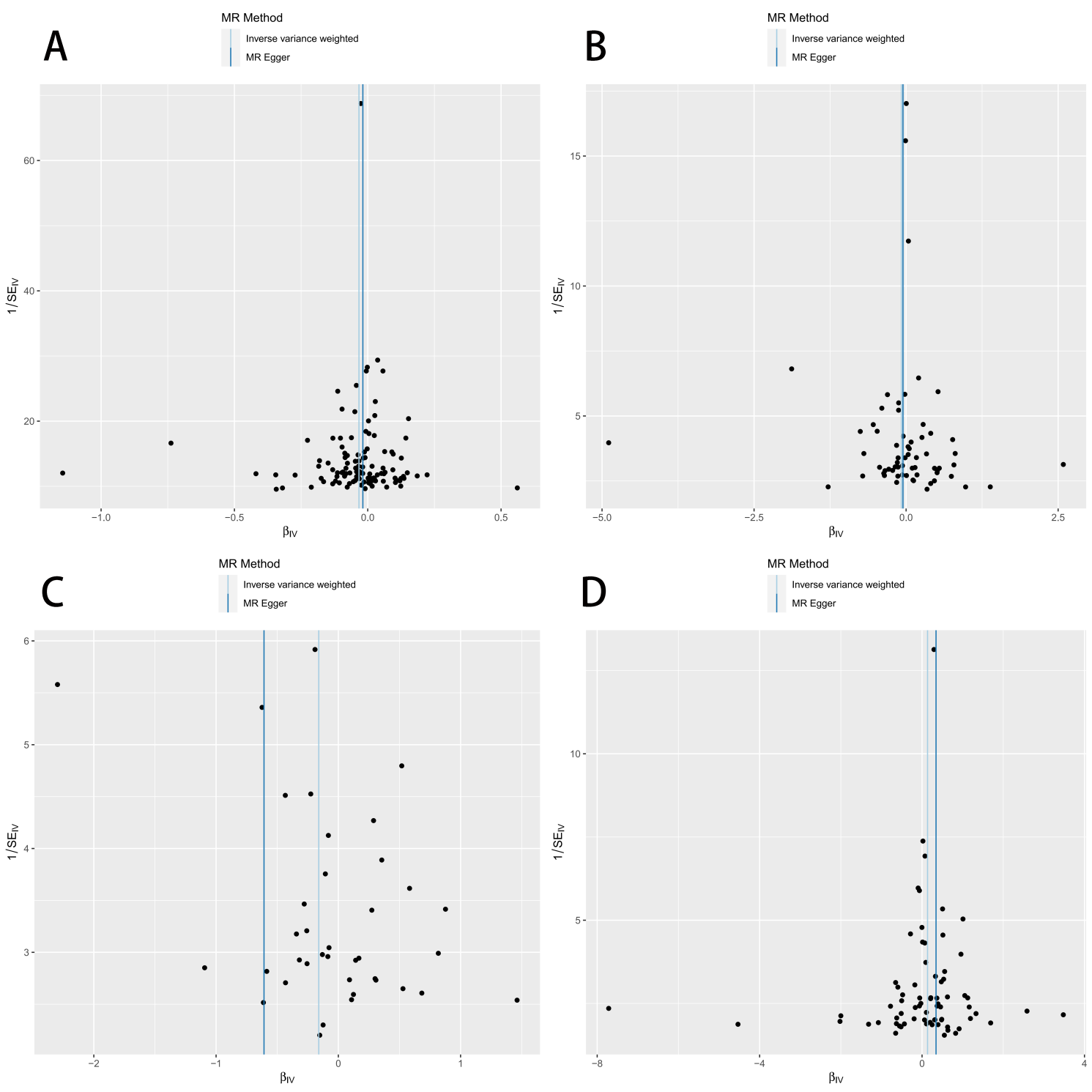


Supplementary Figure 6 Funnel plot: (A) T2D and LA; (B) FBG and LA; (C) FI and LA; (D) HbA1c and LA

Note: Each point on the scatter plot represents an instrumental variable. The x-axis shows the effect size (β value) from MR analysis based on individual SNPs. The y-axis represents the inverse of the standard error, indicating the weight of the SNP. A β value greater than 0 suggests that an increase in the exposure variable will result in an increase in the outcome variable. The colored line depicts the fitted effects from the corresponding MR method.


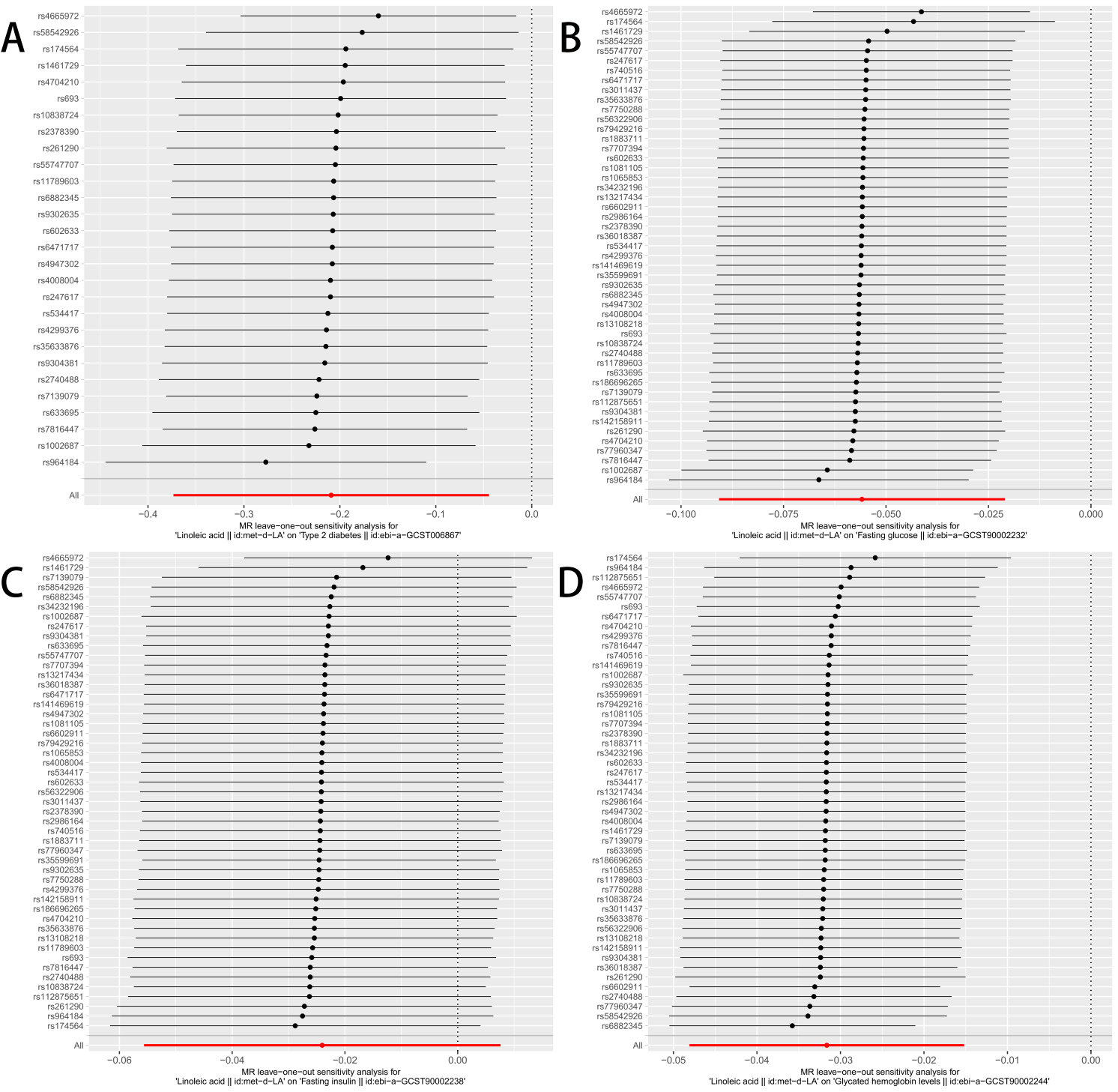


Supplementary Figure 7 Leave-one-out sensitivity analysis:(A) LA and T2D; (B) LA and FBG; (C) LA and FI; (D) LA and HbA1c

Note: This is a graph of the leave-one-out sensitivity analysis. The essence of this analysis is similar to the classical statistical iterative method used when building predictive models. It assesses whether omitting an individual SNP causes a significant change in the outcome. The X-axis represents the MR results' effect (β value), while the Y-axis represents the particular SNP that is excluded.


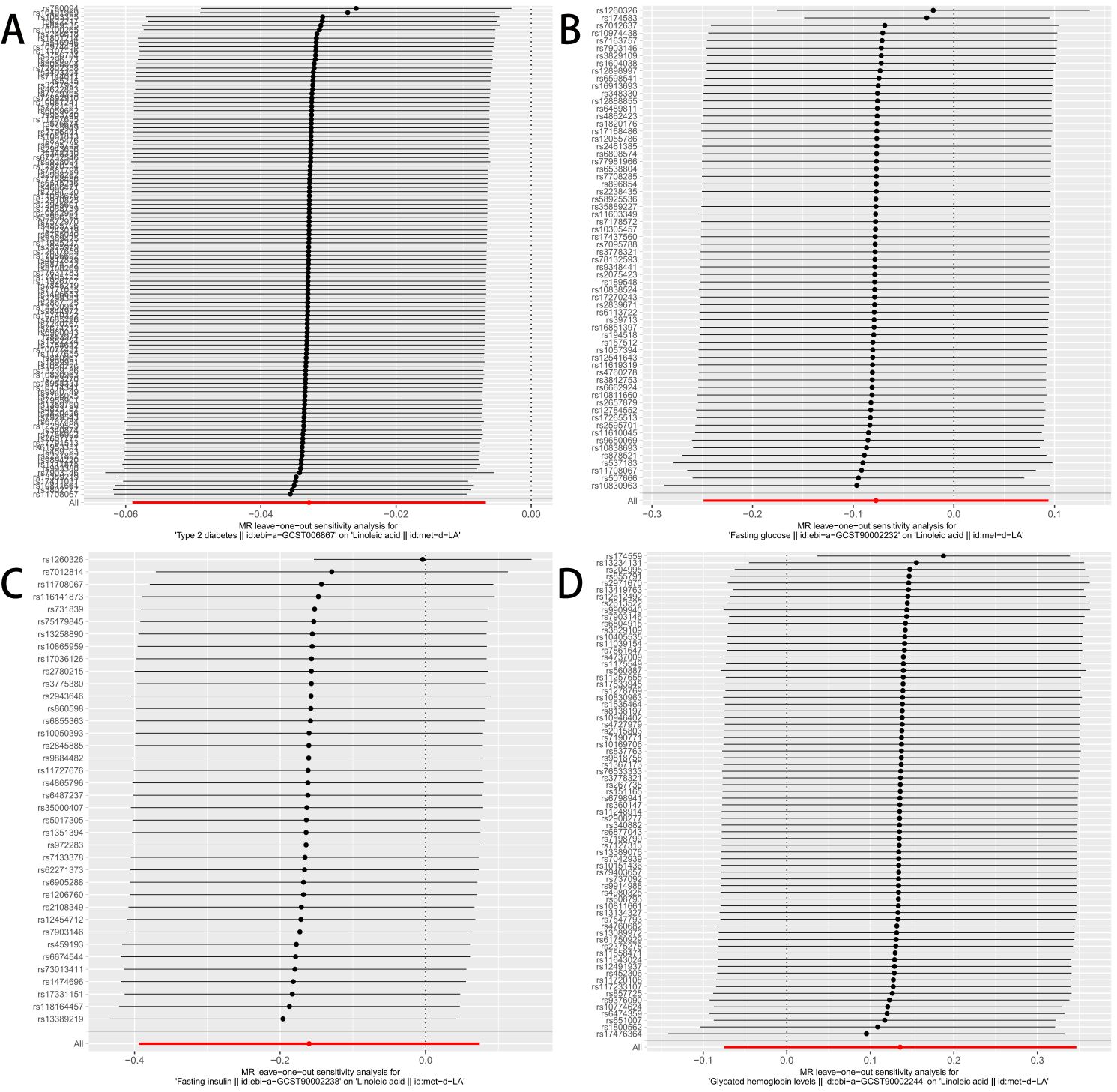


Supplementary Figure 8 Leave-one-out sensitivity analysis:(A) T2D and LA; (B) FBG and LA; (C) FI and LA; (D) HbA1c and LA

Note: This is a graph of the leave-one-out sensitivity analysis. The essence of this analysis is similar to the classical statistical iterative method used when building predictive models. It assesses whether omitting an individual SNP causes a significant change in the outcome. The X-axis represents the MR results' effect (β value), while the Y-axis represents the particular SNP that is excluded.
